# EVI/WLS function is regulated by ubiquitylation and is linked to ER-associated degradation by ERLIN2

**DOI:** 10.1242/jcs.257790

**Published:** 2021-08-18

**Authors:** Lucie M. Wolf, Annika M. Lambert, Julie Haenlin, Michael Boutros

**Affiliations:** 1 Divisionof Signalling and Functional Genomics,German Cancer Research Center (DKFZ), D-69120 Heidelberg, Germany; 2 BioQuant & Department for Cell and Molecular Biology, Medical Faculty Mannheim, Heidelberg University, D-69120 Heidelberg, Germany

**Keywords:** ERAD, Proteolysis, Ubiquitylation, EVI/WLS, Wnt signalling

## Abstract

WNT signalling is important for development in all metazoans and is associated with various human diseases. The ubiquitin–proteasome system (UPS) and regulatory endoplasmic reticulum-associated degradation (ERAD) have been implicated in the production of WNT proteins. Here, we investigated how the WNT secretory factor EVI (also known as WLS) is ubiquitylated, recognised by ERAD components and subsequently removed from the secretory pathway. We performed a focused immunoblot-based RNAi screen for factors that influence EVI/WLS protein stability. We identified the VCP-binding proteins FAF2 and UBXN4 as novel interaction partners of EVI/WLS and showed that ERLIN2 links EVI/WLS to the ubiquitylation machinery. Interestingly, we also found that EVI/WLS is ubiquitylated and degraded in cells irrespective of their level of WNT production. This K11, K48 and K63-linked ubiquitylation is mediated by the E2 ubiquitin-conjugating enzymes UBE2J2, UBE2K and UBE2N, but is independent of the E3 ubiquitin ligases HRD1 (also known as SYVN1) and GP78 (also known as AMFR). Taken together, our study identifies factors that link the UPS to the WNT secretory pathway and provides mechanistic details of the fate of an endogenous substrate of regulatory ERAD in mammalian cells.

This article has an associated First Person interview with the first author of the paper.

## Abstract (precis)

**Summary:** The WNT secretory factor EVI/WLS is ubiquitylated and linked to ER-associated degradation by multiple proteins, providing insight into the link between WNT signalling and the ubiquitin–proteasome system.

## INTRODUCTION

Cell–cell communication is fundamental to multicellular organisms and as such requires tight and nuanced regulation on many levels. Protein stability and turnover are essential to guarantee flexible and context-dependent cellular responses to signalling cues, together with various other mechanisms. Eukaryotic protein degradation is mediated by two main systems: the ubiquitin–proteasome system (UPS) and the autophagy–lysosomal pathway, which can both be initiated by tagging substrates with the small protein ubiquitin (Pohl and Dikic, 2019). The specificity of this posttranslational modification relies on the subsequent action of three different enzymatic processes, namely the activation of ubiquitin by the enzyme E1, followed by transfer of ubiquitin to an E2 ubiquitin-conjugating enzyme and then to the target polypeptide with the help of an E3 ubiquitin ligase (Swatek and Komander, 2016). Whereas substrate recognition is the main task of the numerous E3 ubiquitin ligases, only ∼35 mammalian E2 ubiquitin-conjugating enzymes regulate the initial priming with single ubiquitin moieties and the formation of polyubiquitin chains (Deol et al., 2019; van Wijk et al., 2009; Weber et al., 2016). Since ubiquitin itself has eight potential acceptor sites for further ubiquitin modifications [at lysine (K)6, K11, K27, K29, K33, K48, K63 and at the N-terminal methionine], the resulting chains can be of variable geometry and length (Clague et al., 2015; Deol et al., 2019). K48 and K63 ubiquitin linkages are the best studied and most common types, and they are primarily functionally involved in proteasomal degradation (K48) or proteasome-independent processes (K63), such as endosomal trafficking and targeting to the lysosomes (Akutsu et al., 2016; Clague et al., 2015; Erpapazoglou et al., 2014; Swatek and Komander, 2016).

A major role for the UPS is to remove terminally misfolded proteins to prevent their aggregation and potential harm to the cell, even from within the endoplasmic reticulum (ER). ER-associated degradation (ERAD) recognises, extracts, ubiquitylates and delivers ER membrane proteins or proteins within the secretory route to the proteasome (Bhattacharya and Qi, 2019; Christianson and Ye, 2014; Lopata et al., 2020; Sun and Brodsky, 2019). Several ER membrane-resident E3 ubiquitin ligases provide the polyubiquitin signal for ERAD, most notably HRD1 (also known as SYVN1), GP78 (also known as AMFR), and MARCH6 (also known as MARCHF6), which have well-studied orthologues in yeast (Lopata et al., 2020; Preston and Brodsky, 2017). Another major protein in this process is the AAA ATPase VCP (also known as p97; orthologue of yeast Cdc48), which uses ATP to extract substrates from the ER or its membrane into the cytoplasm and can be recruited to the ER membrane by proteins with a VCP-binding domain, such as FAF2 and UBXN4 (Bodnar and Rapoport, 2017; Meyer and Weihl, 2014).

In addition to protein quality control, ERAD can also impact cellular signalling by regulating the availability of mature proteins through quantity control (Bhattacharya and Qi, 2019; Hegde and Ploegh, 2010; Printsev et al., 2017). It is assumed that ERAD quality and quantity control are mechanistically similar and differ mostly during substrate recognition (Hegde and Ploegh, 2010). While misfolded proteins can be recognised by exposed hydrophobic patches or prolonged retention within the ER, the selection of properly folded and functional proteins for degradation is less straightforward and has only been investigated in detail for a few mammalian substrates. Indeed, only ∼20–30 endogenous substrates for mammalian regulatory ERAD have been reported, and for most it is unclear how they are recognised, ubiquitylated and linked to the ERAD machinery (Bhattacharya and Qi, 2019; Printsev et al., 2017). Furthermore, various studies have focused on ubiquitylation by HRD1, and other ER membrane resident E3 ubiquitin ligases remain poorly defined (Fenech et al., 2020).

**Fig. 1.**
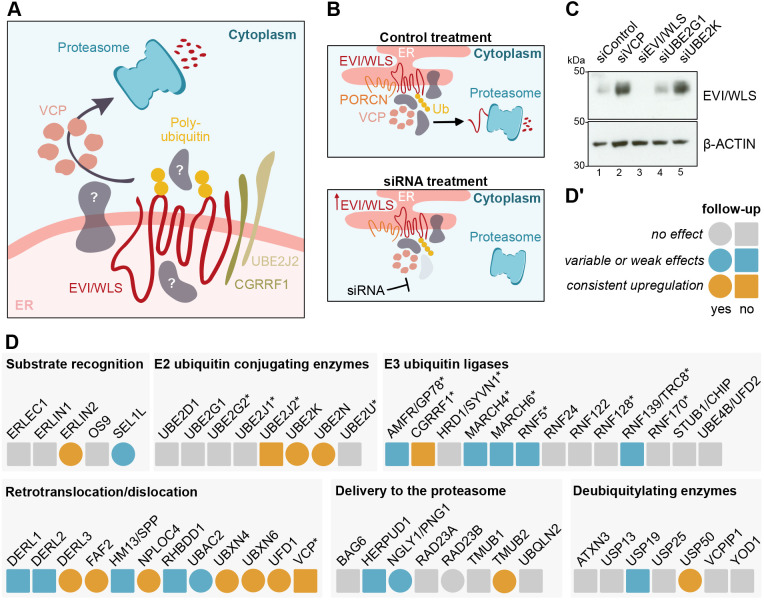
**siRNA-based low-throughput screen identifies novel candidates involved in the degradation of EVI/WLS.** (A) Schematic representation of the current understanding of the ERAD of EVI/WLS, and open questions. EVI/WLS is ubiquitylated by CGRRF1 and UBE2J2 before being extracted from the ER membrane with the help of VCP and degradation by the proteasome. (B) Schematic illustration of the principle behind the screening procedure using siRNAs. EVI/WLS protein accumulates if the siRNA targets a mRNA encoding a protein important for EVI/WLS degradation (Ub, polyubiquitin chain). (C) EVI/WLS protein levels were analysed after siRNA-mediated knockdown of target genes. Increased EVI/WLS protein levels compared to siControl treatment indicated the possible involvement of the candidate in ERAD of EVI/WLS. HEK293T cells were harvested 72 h after transfection with the indicated siRNAs. β-actin served as loading control. Western blots are representative of three independent experiments. (D,D′) Results of the siRNA-based low-throughput screen. Candidates that had no effect are marked in grey, candidates that had variable or weak effects are marked in blue and candidates that showed a strong and consistent upregulation of EVI/WLS levels are marked in orange. Circles indicate follow-up experiments. Asterisks indicate genes that were previously tested in Glaeser et al. (2018). A detailed table, including gene accession numbers and phenotypes in HEK293T and A375 cells, can be found in Table S1. The western blots underlying this analysis are shown in Fig. S1.

Protein stability is at the heart of many cellular communication pathways, and it is not surprising that signalling cascades have numerous intersections with the UPS. An example is the regulated degradation of β-catenin in the absence of active WNT signalling (Aberle et al., 1997) – a pathway that controls embryonic growth and patterning and ensures tissue homeostasis in adults, and whose deregulation can lead to cancer (Nusse and Clevers, 2017; Zhan et al., 2017). In line with these findings, we have recently demonstrated that the conserved transmembrane protein EVI (also known as WLS) is a target of regulatory ERAD and is ubiquitylated by the E2 ubiquitin-conjugating enzyme UBE2J2 and the E3 ubiquitin ligase CGRRF1, before being removed from the ER with the help of VCP (Glaeser et al., 2018). EVI/WLS degradation is inhibited by binding to WNT ligands, which are modified with palmitoleate by the acyl-transferase porcupine (PORCN; Takada et al., 2006). However, it remains unclear how EVI/WLS is linked to the ubiquitylation machinery and how VCP is recruited, as EVI/WLS itself has no VCP interaction domain (Fig. 1A). Furthermore, knockdown of UBE2J2 and/or CGRRF1 does not completely abolish EVI/WLS ubiquitylation, indicating the involvement of additional, currently unknown E2 and/or E3 enzymes (Glaeser et al., 2018). In this context, ERAD of EVI/WLS seems to be especially interesting due to its independence of the well-studied ERAD-associated E3 ubiquitin ligases HRD1, GP78 and MARCH6.

In this study, we performed a focused RNAi- and western blot-based screen of EVI/WLS protein abundance to identify factors regulating stability of the protein. We found that EVI/WLS degradation is initiated by interaction with ERLIN2, which precedes EVI/WLS ubiquitylation. Thereafter, ubiquitylated EVI/WLS is linked to VCP by its interaction with FAF2 and UBXN4. Furthermore, we demonstrate that human EVI/WLS is additionally ubiquitylated by UBE2N and UBE2K (as well as UBE2J2) and is modified with ubiquitin of various linkage types (K11, K48 and K63), which has important consequences for protein stability and function. Using melanoma cells, which express high levels of WNT ligands, we show that endogenous EVI/WLS proteins are ubiquitylated and degraded even in the presence of WNTs. Thus, our data provide important insights into the mechanism of the recognition and degradation of EVI/WLS, an endogenous mammalian substrate of regulatory ERAD, and further emphasises the link between ubiquitylation and WNT signalling.

## RESULTS

### A focused screen for ERAD candidate genes

Several proteins are involved in the recognition and retrotranslocation or dislocation of ERAD substrates, but additional regulators of EVI/WLS remain elusive. To address this in a systematic manner, we performed a focused RNAi- and western blot-based screen in HEK293T cells. As readout we used the EVI/WLS protein level after treatment with a pool of four siRNAs, and increased protein levels indicated impaired degradation (Fig. 1B). In total, we tested 53 candidates, including those previously tested by Glaeser et al. (2018) (Fig. 1C,D; Fig. S1, Table S1). Of the 53 candidates, 21 were either E2 ubiquitin-conjugating enzymes or E3 ubiquitin ligases and seven were deubiquitylases. Furthermore, we included five proteins important for substrate recognition within the ER and eight proteins involved in delivery of the substrate to the proteasome in the cytoplasm. The last group of candidates consisted of 12 proteins associated with substrate retrotranslocation or dislocation by either forming a channel, cleaving the substrate, or by recruiting or interacting with VCP. In follow-up experiments, we investigated 15 selected candidates in more detail by assessing the effects of the respective single siRNAs on EVI/WLS protein and, in selected cases, on mRNA level. Reverse transcription (RT)-qPCR was used to analyse knockdown efficiencies and to exclude transcriptional regulation of EVI/WLS, thus highlighting posttranslational effects. siRNA targeting EVI/WLS (siEVI/WLS) was used as an on-target control and siRNA targeting VCP (siVCP) was used as a positive control. siVCP has been previously shown to increase endogenous EVI/WLS protein levels without affecting *EVI/WLS* mRNA (Glaeser et al., 2018). The silencing of mRNA and protein expression by siEVI/WLS and siVCP were efficient (Figs S1–S3), and VCP downregulation induced upregulation of EVI/WLS protein levels, as described previously (Glaeser et al., 2018). The majority of the tested siRNAs efficiently reduced mRNA levels to values between 5% and 20% of the control without affecting EVI/WLS protein expression. However, ten candidates (DERL3, NGLY1, NPLOC4, RAD23B, SEL1L, TMUB2, UBAC2, UBXN6, UFD1 and USP50) were not followed up further due to variation in the western blot experiments between different single siRNAs and biological replicates indicating non-target effects of the siRNAs (Figs S2,S3).

### EVI/WLS protein levels are regulated by the candidate proteins

**Fig. 2.**
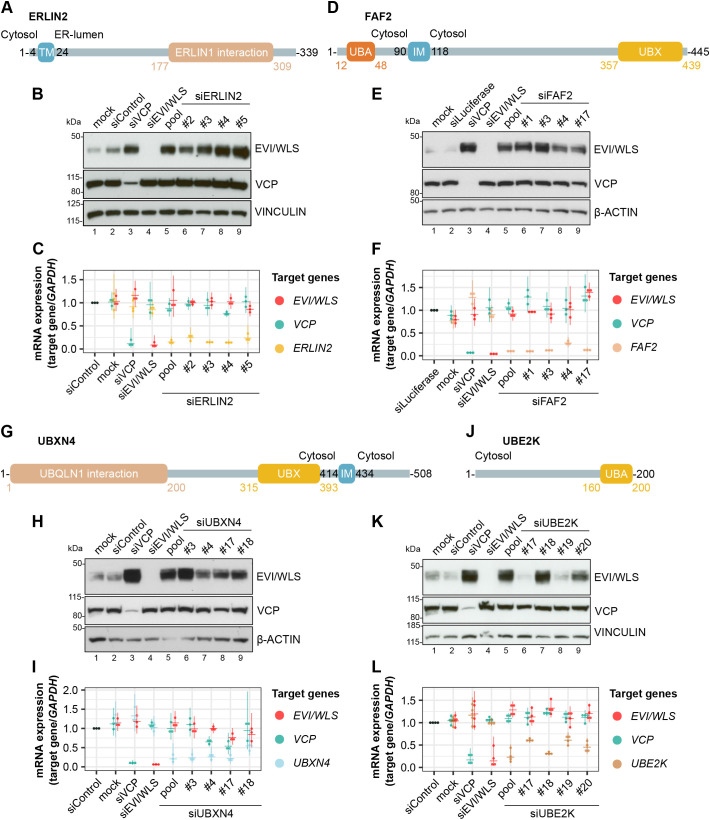
**ERLIN2, FAF2, UBXN4 and UBE2K regulate endogenous EVI/WLS at the protein level.** (A,D,G,J) Schematic representations of the proteins ERLIN2 (A), FAF2 (D), UBXN4 (G) and UBE2K (J) according to UniProt identifiers O94905, Q96CS3, Q92575 and P61086, respectively. Numbers indicate amino acid positions. IM, intramembrane domain; TM, transmembrane domain; UBA, ubiquitin-associated domain important for binding to ubiquitin; UBX, ubiquitin regulatory X domain important for binding to VCP. (B,C,E,F,H,I,K,L) Knockdown of ERLIN2 (B,C), FAF2 (E,F), UBXN4 (H,I) or UBE2K (K,L) increased EVI/WLS protein levels (B,E,H,K) but had no effect on *EVI/WLS* mRNA expression (C,F,I,L). HEK293T cells were harvested 72 h after transfection of the indicated siRNAs. siRNAs targeting *ERLIN2, FAF2* or *UBXN4* were used as either single siRNAs (numbered) or an equimolecular mix of all four respective siRNAs (pool). siRNA numbers are according to the manufacturer product code. Mock-transfected cells were included as a control. Vinculin (B,K) or β-actin (E,H) served as loading controls. Western blots are representative of three independent experiments. In C,F,I,L, target gene expression was normalised to siControl treatment, and *GAPDH* served as reference gene. Individual data points of three or four independent experiments with mean and 95% confidence intervals are shown.

Five genes from our screen (*FAF2*, *ERLIN2*, *UBXN4*, and the E2 ubiquitin-conjugating enzymes *UBE2K* and *UBE2N*) were selected for further analysis due to their consistent upregulation of EVI/WLS protein levels without changes in *EVI/WLS* mRNA levels (Figs 2 and 4A,B).

**Fig. 3.**
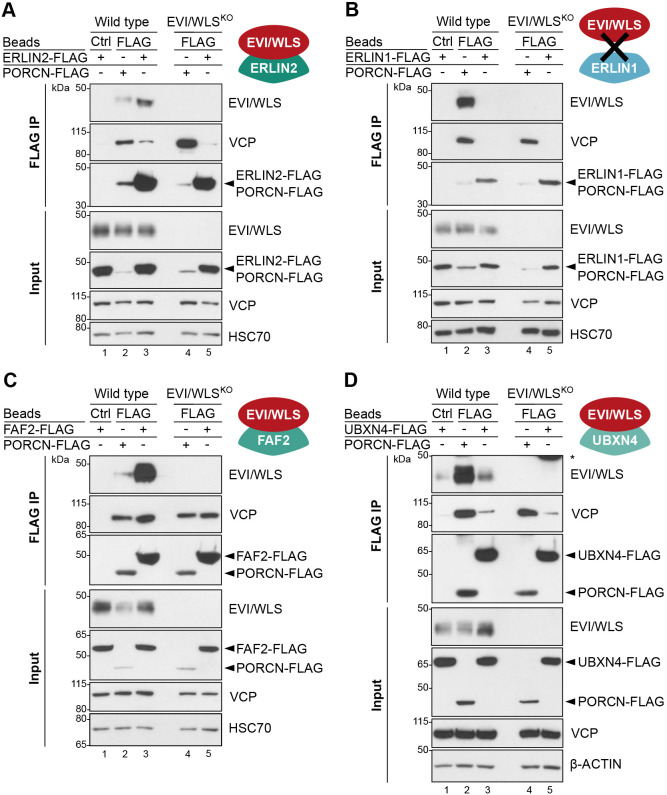
**ERLIN2–FLAG, FAF2–FLAG and UBXN4–FLAG interact with endogenous EVI/WLS.** (A–D) Immunoprecipitation (IP) experiments confirmed interaction between endogenous EVI/WLS and ERLIN2–FLAG (A), FAF2–FLAG (C) and UBXN4–FLAG (D) but not ERLIN1–FLAG (B). Wild-type and EVI/WLS knockout (EVI/WLS^KO^) HEK293T cells were transfected with ERLIN1–FLAG, ERLIN2–FLAG, FAF2–FLAG, UBXN4–FLAG or PORCN–FLAG overexpression plasmids, as indicated. After 48 h, total cell lysates were sampled for input control (∼15 µg of total lysate) or used for IP with anti-FLAG or control (Ctrl) beads to precipitate FLAG-tagged proteins and their interaction partners, detected by western blotting for the indicated proteins. HSC70 (also known as HSPA8) or β-actin served as loading control. Western blots are representative of three independent experiments.

To test whether these proteins bind to EVI/WLS, we performed immunoprecipitation studies. Here, we generated FLAG-tagged overexpression constructs of ERLIN2, FAF2, UBXN4 and UBE2K to investigate their interaction with endogenous EVI/WLS and VCP. PORCN–FLAG was used as a positive control because it has been previously shown to bind to EVI/WLS and VCP (Glaeser et al., 2018). Indeed, our immunoprecipitation studies demonstrate that endogenous EVI/WLS interacts with ERLIN2–FLAG, FAF2–FLAG, and UBXN4–FLAG (Fig. 3A,C,D, respectively). Furthermore, we detected an interaction between PORCN–FLAG and endogenous ERLIN2 and FAF2 (Fig. S4A,B). We also confirmed previously described interactions within the ERAD machinery, such as between FAF2 and ERLIN2 (Fig. S4A,B; Christianson et al., 2012), and between VCP and the UBX domain-containing proteins FAF2 and UBXN4 (Fig. 3C,D; Schuberth and Buchberger, 2008), but interestingly not between FAF2 and UBXN4 (Fig. S4A). Importantly, immunoprecipitation of ERLIN2 or FAF2 without overexpression revealed interaction between both endogenous proteins and endogenous EVI/WLS (Fig. S4C). ERLIN1 is an important interaction partner of ERLIN2, but it did not influence EVI/WLS protein levels in our screen (Fig. 1D; Fig. S1). Accordingly, our pulldown experiments revealed that ERLIN1 interacts with endogenous ERLIN2, but not with EVI/WLS or VCP (Fig. 3B; Fig. S4B).

Our screen also identified two E2 ubiquitin-conjugating enzymes, UBE2K and UBE2N, which regulate EVI/WLS protein levels. However, we were unable to detect an interaction between FLAG–UBE2K and EVI/WLS in immunoprecipitation experiments (Fig. S4D), presumably due to the transient nature of the interaction and the stringent pulldown conditions used in this study.

**Fig. 4.**
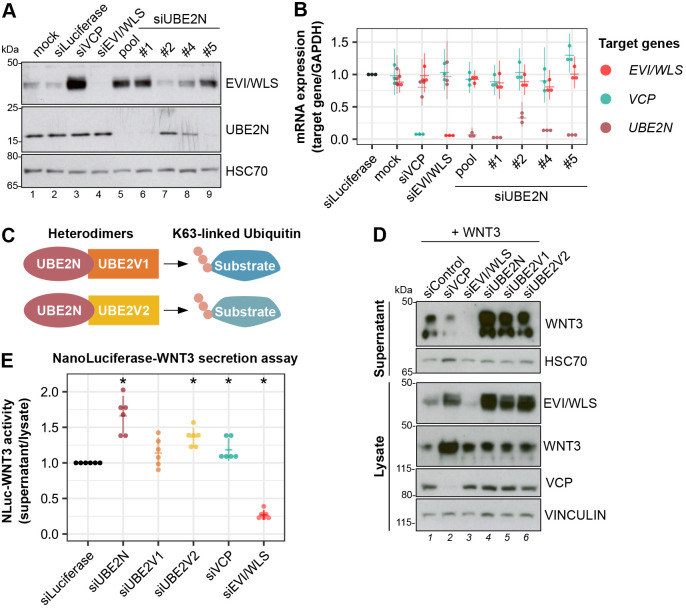
**UBE2N and UBE2V2 regulate EVI/WLS protein levels and WNT secretion.** (A) Knockdown of UBE2N increased EVI/WLS protein levels. HEK293T cells were harvested 72 h after transfection with the indicated siRNAs or mock transfection. siRNAs targeting *UBE2N* were used as either single siRNAs (numbered) or an equimolecular mix of all four respective siRNAs (pool). HSC70 served as loading control for the western blots. (B) mRNA expression analyses showed mostly potent gene silencing by pooled or single siRNAs, with little effect on other investigated mRNAs. HEK293T cells were harvested 72 h after transfection with the indicated siRNAs or mock transfection. Each mRNA was targeted by either single siRNAs or an equimolecular mix of all four respective siRNAs (pool) to analyse their effect on mRNA expression. Target gene expression was normalised to expression in siLuciferase-treated cells, and *GAPDH* served as a reference gene. Individual data points from three independent experiments with mean and 95% confidence intervals are shown. (C) Schematic representation of how UBE2N forms active complexes with UBE2V1 or UBE2V2 to modify substrates with K63-linked ubiquitin. (D) Knockdown of UBE2N, UBE2V1 and UBE2V2 in combination with WNT3 overexpression increased EVI/WLS protein levels and WNT secretion compared to control treatment. HEK293T cells were transfected with the indicated siRNAs. 24 h after siRNA transfection, cells were additionally transfected with WNT3 plasmid. Culture supernatants and cell lysates were analysed by western blotting using antibodies against the indicated proteins. Vinculin and HSC70 served as loading controls for the lysate and supernatant, respectively. (E) NanoLuciferase (NLuc)–WNT3 secretion was elevated after knockdown of UBE2N. HEK293T cells were transfected with the indicated siRNAs and additionally transfected with NLuc–WNT3 and firefly luciferase 24 h later. 48 h later, NLuc activity was determined in the cell supernatant and normalised to NLuc and firefly activity in the cell lysates. Data points derived from six independent experiments with mean and 95% confidence intervals are shown. *P=0.03125 (one-sample Wilcoxon Signed Rank test, two-sided). Western blots in A and D are representative of three independent experiments.

RNAi-induced knockdown of UBE2N increased EVI/WLS protein levels in HEK293T cells proportional to the siRNA efficiency (Fig. 4A,B). UBE2N (also known as UBC13) is an E2 ubiquitin-conjugating enzyme with K63 linkage specificity required for protein localisation and endosomal trafficking (Akutsu et al., 2016; Erpapazoglou et al., 2014; Swatek and Komander, 2016), processes which are also essential for the function of EVI/WLS. In addition to UBE2N, we also tested the effect of the catalytically inactive UBE2N interaction partners UBE2V1 and UBE2V2 (Andersen et al., 2005; McKenna et al., 2001) on EVI/WLS protein levels (Fig. 4). Importantly, knockdown of UBE2N and its interaction partners not only increased EVI/WLS protein levels, but also increased the secretion of WNT ligands in HEK293T cells upon WNT3 or NanoLuciferase–WNT3 overexpression, indicating a possible modulatory effect on WNT signalling in general (Fig. 4D,E). Indeed, Zhang et al. have described the importance of the UBE2N orthologue UBC-13 for WNT-dependent processes in worms (Zhang et al., 2018). The effects of UBE2V1 knockdown on protein stability and WNT secretion were more variable and suggested indirect effects, maybe via stability of the UBE2N complex (Fig. 4D,E). Under these conditions, secretion of WNT3 was not increased after knockdown of VCP (Fig. 4D,E).

### EVI/WLS is ubiquitylated and degraded in the presence of endogenous WNT ligands

**Fig. 5.**
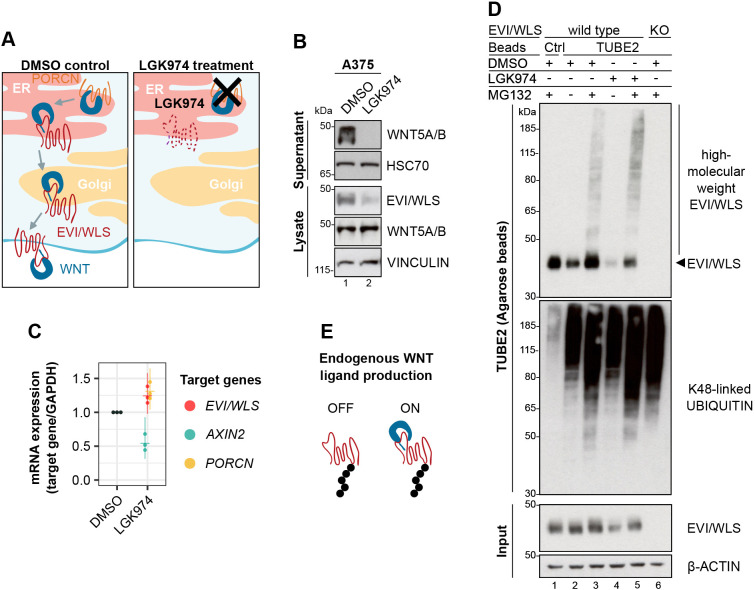
**EVI/WLS is ubiquitylated in cells with endogenous WNT ligands.** (A) Schematic illustration of the mode of action of LGK974. LGK974 prevents WNT ligands from being lipid-modified in the ER by inhibiting the acyl-transferase PORCN. Unlipidated WNTs cannot associate with EVI/WLS and are not secreted from the WNT producing cell. (B,C) LGK974 treatment reduced intracellular EVI/WLS levels and abolished the secretion of WNT5A and/or WNT5B (WNT5A/B) without reducing EVI/WLS gene expression. A375 melanoma cells were treated with LGK974 (10 µM) or DMSO for 96 h with daily medium changes. (B) Secreted proteins were precipitated from the supernatant using Blue Sepharose, and relative WNT5A/B levels were compared to those in cell lysates. Vinculin and HSC70 served as loading controls. (C) Target gene expression was normalised to DMSO treatment and *GAPDH* served as reference gene. Individual data points from three independent experiments are shown with mean and 95% confidence intervals. (D) Ubiquitylated EVI/WLS accumulated after inhibition of the proteasome, independent of LGK974 treatment. Wild-type and EVI/WLS knockout (KO) A375 melanoma cells were treated with LGK974 (10 µM) or DMSO for 96 h with daily medium changes. Samples were treated with the proteasome inhibitor MG132 (1 µM), as indicated, 24 h before harvesting. Then, total cell lysates were sampled for input control (∼6.5 µg of total lysate) or used for TUBE2 (agarose) pulldown to precipitate polyubiquitylated proteins. Ubiquitin non-binding control (Ctrl) agarose beads showed a level of unspecific binding, and EVI/WLS^KO^ cells confirmed specificity for EVI/WLS. β-actin served as loading control. (E) EVI/WLS is ubiquitylated in the presence (ON) and absence (OFF) of endogenous WNT ligands. Western blots in B and D are representative of three independent experiments.

HEK293T cells are commonly used as a model to analyse WNT signalling due to their low endogenous WNT secretion. The striking observation that ubiquitylation of EVI/WLS might influence WNT ligand secretion indicates that it might regulate WNT signalling itself. Hence, we were intrigued by the ubiquitylation status of EVI/WLS in cells with high endogenous WNT ligand production. Melanoma is a skin cancer with poor prognosis in advanced stages (Schadendorf et al., 2018), and tumour progression and metastasis are associated with the expression of non-canonical WNT ligands (Webster et al., 2015). For this reason, we chose the melanoma cell line A375 for further studies. This cell line has been shown to express high amounts of WNT proteins, most notably WNT5A (Yang et al., 2012). Because the overexpression of WNT ligands in HEK293T cells leads to the stabilisation of EVI/WLS protein (Glaeser et al., 2018), we wanted to investigate whether the effect can be reversed in A375 cells by using LGK974, an inhibitor of the acyl-transferase PORCN, thus preventing the secretion of WNT ligands. As expected, LGK974 treatment abolished the secretion of WNT5A and reduced EVI/WLS protein levels in the cell lysates without affecting *EVI/WLS* mRNA expression (Fig. 5A–C; note that the antibody used in these studies recognises both WNT5A and WNT5B).

Next, we were interested in the ubiquitylation status of EVI/WLS on an endogenous level. For this reason, we used tandem ubiquitin-binding entities (TUBEs) to enrich for ubiquitylated proteins followed by western blot analysis of denatured protein samples. Combinatorial treatment with LGK974 and the proteasome inhibitor MG132 increased the accumulation of high molecular weight bands of EVI/WLS, as expected. Surprisingly, treatment with MG132 and dimethyl sulfoxide (DMSO; used as a vehicle control for LGK974) resulted in the accumulation of ubiquitylated EVI/WLS as well, indicating that EVI/WLS is ubiquitylated in the absence and presence of lipid-modified endogenous WNT ligands (Fig. 5D,E). These results show that EVI/WLS protein levels depend on the availability of mature WNT ligands in cells with high endogenous WNT signalling, but a certain fraction of the EVI/WLS protein is ubiquitylated and targeted for degradation even in the presence of WNT ligands. This fraction probably includes misfolded EVI/WLS, which is targeted by qualitative ERAD. To identify genes that mediate the degradation of EVI/WLS in A375 cells, we silenced candidate genes using RNAi in the presence of LGK974 and then analysed EVI/WLS protein levels by western blotting. Among the tested genes, the knockdown of UBE2J2, CGRRF1 and VCP consistently elevated EVI/WLS protein levels, in line with our results in HEK293T cells (Fig. S5A–F; Glaeser et al., 2018). Although it has been reported previously that EVI/WLS influences the proliferation of melanoma cells (Yang et al., 2012), siRNA-mediated knockdown of EVI/WLS did not change the proliferation of A375 cells compared to that of control cells transfected with a control siRNA targeting luciferase (siLuciferase) within the timespans relevant for our experiments (up to 96 h; Fig. S5G). However, transfection of either siVCP or siRNA targeting UBE2N (siUBE2N) reduced A375 proliferation and affected cell viability (Fig. S5G), as expected due to the broad range of essential functions described for both proteins.

### ERLIN2 connects EVI/WLS to the ubiquitylation machinery

Our results so far demonstrate that ERLIN2, FAF2, UBXN4, UBE2K and UBE2N can regulate EVI/WLS protein levels and that EVI/WLS is stabilised in cells with endogenously active WNT signalling but can still be ubiquitylated and degraded. However, it remained elusive how these candidates can influence the ubiquitylation of EVI/WLS.

**Fig. 6.**
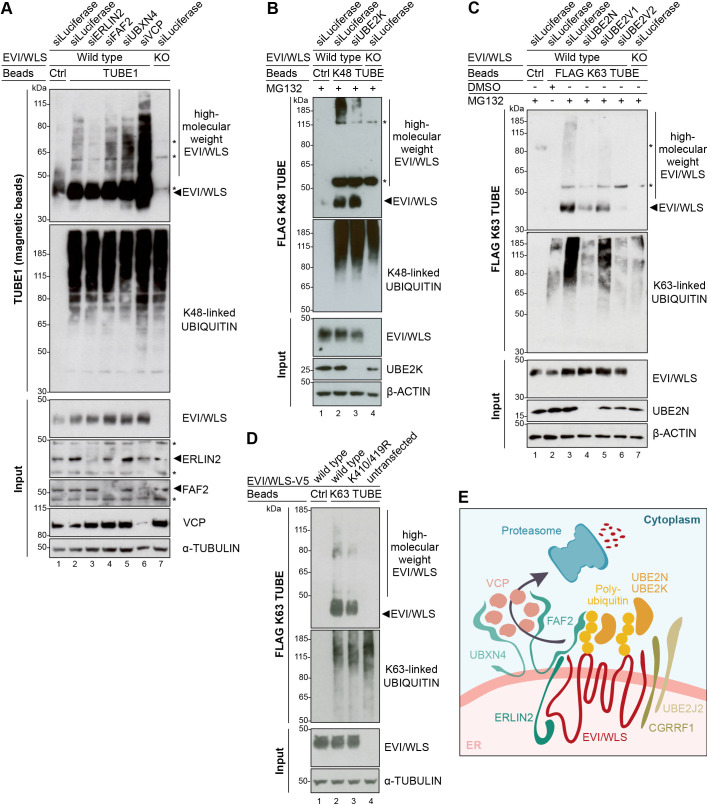
**ERLIN2 links EVI/WLS to the ubiquitylation machinery.** (A) Knockdown of ERLIN2 reduced the ubiquitylation of EVI/WLS, while knockdown of FAF2 and UBXN4 increased it. Wild-type and EVI/WLS knockout (KO) A375 melanoma cells were harvested 72 h after transfection with the indicated siRNAs. Then, total cell lysates were sampled for input control (∼6.5 µg of total lysate) or used for TUBE1 pulldown to precipitate polyubiquitylated proteins. (B) FLAG K48-specific TUBE pulldown confirmed that EVI/WLS is modified with K48-linked ubiquitin chains by UBE2K. Wild-type and EVI/WLS KO A375 melanoma cells were harvested 72 h after transfection with the indicated siRNAs. Samples were treated with the proteasome inhibitor MG132 (1 µM) 24 h before harvesting. Total cell lysates were sampled for input control (∼6.5 µg of total lysate) or used for FLAG K48-specific TUBE pulldown to specifically precipitate proteins modified with K48-linked polyubiquitin. (C) FLAG K63-specific TUBE pulldown confirmed that EVI/WLS is modified with K63-linked ubiquitin chains by UBE2N. Wild-type and EVI/WLS KO A375 melanoma cells were harvested 72 h after transfection with the indicated siRNAs. Samples were treated with the proteasome inhibitor MG132 (1 µM) 24 h before harvesting. Total cell lysates were sampled for input control (∼6.5 µg of total lysate) or used for FLAG K63-specific TUBE pulldown to specifically precipitate proteins modified with K63-linked polyubiquitin. (D) FLAG K63-specific TUBE pulldown confirmed that the positions K410 and/or K419 of EVI/WLS are important for K63-linked ubiquitylation. Wild-type or K410R K419R double mutant (K410/419R) EVI/WLS–V5 constructs were overexpressed in A375 melanoma EVI/WLS KO cells and harvested 48 h later. Total cell lysates were sampled for input control (∼13 µg of total lysate) or used for FLAG K63-specific TUBE pulldown to specifically precipitate proteins modified with K63-linked polyubiquitin. (E) Schematic representation of the ERAD of EVI/WLS. EVI/WLS is recognised by ERLIN2 and ubiquitylated by CGRRF1 and UBE2J2 as well as UBE2K and UBE2N. The latter adds K63-linked ubiquitin chains to EVI/WLS. Ubiquitylated EVI/WLS binds FAF2 and UBXN4, which recruit VCP to the ER membrane and mediate the delivery of EVI/WLS to the proteasome. In A–C, asterisks mark nonspecific signals. Ubiquitin non-binding control (Ctrl) agarose or magnetic beads showed level of unspecific binding, and EVI/WLS KO cells confirmed specificity for EVI/WLS. Western blots in A–D are representative of three independent experiments. α-tubulin and β-actin served as loading controls, as indicated.

To determine whether ERLIN2, FAF2 and UBXN4 are required for EVI/WLS ubiquitylation, we performed precipitations using pan-ubiquitin-specific TUBE1 coupled to magnetic beads in combination with RNAi-mediated knockdown of these candidates and analysed denatured protein samples using western blotting. Knockdown of ERLIN2 led to a reduction of high molecular weight EVI/WLS bands, whereas knockdown of FAF2 and UBXN4 resulted in an increase of polyubiquitylated EVI/WLS (Fig. 6A). Taken together, this data suggests that ERLIN2 functions as a bridge connecting EVI/WLS to the ubiquitylation machinery and that FAF2 and UBXN4 interact with EVI/WLS after it is ubiquitylated, but before it is delivered to the proteasome. We used siVCP as a positive control, which showed the strongest accumulation of high molecular weight EVI/WLS bands compared to all conditions (Fig. 6A), presumably indicating that EVI/WLS is subject to several parallel recruitment mechanisms that all culminate in dislocation by VCP.

It is known that EVI/WLS is ubiquitylated by UBE2J2 and CGRRF1 (Fenech et al., 2020; Glaeser et al., 2018) and that the UBE2J2 orthologue in yeast (Ubc6) is indispensable for priming a broad range of substrates with monoubiquitin or K11-linked ubiquitin dimers (Weber et al., 2016; Xu et al., 2009). However, so far, the kind of ubiquitin linkage types present on EVI/WLS and the sites of the modifications have not been identified, since the TUBEs used to detect ubiquitylation shown in Figs 5D and 6A were non-selective and unable to distinguish between different ubiquitin linkage types. Therefore, we used mutant ubiquitin constructs that only allow one linkage type, either K11, K48 or K63 (referred to here as K11 ubiquitin, K48 ubiquitin and K63 ubiquitin, respectively; Clague et al., 2015; Tsuchiya et al., 2018; Xu et al., 2009), and analysed denatured protein samples using western blotting. Intriguingly, we detected ubiquitylated EVI/WLS bands upon overexpression of HA-tagged wild-type, K11, K48 or K63 ubiquitin, but not in control conditions (Fig. S6A), indicating the presence of multiple linkage types on EVI/WLS. This data also supports the hypothesis that EVI/WLS is modified by multiple E2 ubiquitin-conjugating enzymes with different linkage type specificities. We hypothesise that UBE2K and UBE2N modify EVI/WLS with K48- and K63-linked ubiquitin, respectively (Fig. S6A).

Linkage-type specific TUBEs can detect differences in K48- or K63-linked ubiquitylation, especially if the substrate protein is modified in parallel with several poly- or mono-ubiquitins and if differences in overall ubiquitylation are small. Therefore, we used linkage-type specific TUBEs and western blotting of denatured protein samples to analyse the linkage types present on EVI/WLS. UBE2K can synthesise K48-linked ubiquitin chains (Chen and Pickart, 1990; Middleton and Day, 2015), and accordingly, knockdown of UBE2K resulted in reduced K48-ubiquitylated EVI/WLS (Fig. 6B). Similarly, silencing of UBE2N and UBE2V2 strongly reduced endogenous high molecular weight EVI/WLS bands after K63-specific pulldown (Fig. 6C). This indicates that UBE2K mediates K48-linked ubiquitylation of EVI/WLS, whereas UBE2N and UBE2V2 mediate K63-linked ubiquitylation of EVI/WLS in human cells. The exact ubiquitylation sites of EVI/WLS remain elusive, and mutational studies to investigate which residue is ubiquitylated by which enzyme are hindered by functional redundancy between the involved E2 enzymes and a large number of potential ubiquitin acceptor sites. Nevertheless, two lysine residues within the third intracellular loop of EVI/WLS are good candidates for ubiquitylation, as they are exposed and potentially easy to reach by interacting proteins (Nygaard et al., 2021). Indeed, EVI/WLS–V5 overexpression constructs in which these lysines K410 and K419 were mutated to arginine showed reduced ubiquitylation after pulldown with K63-specific TUBEs (Fig. 6D).

### EVI/WLS interacts with VCP and FAF2 at the ER

In cells that actively secrete WNTs, EVI/WLS chaperones WNT transport from the ER to the plasma membrane (Bänziger et al., 2006; Bartscherer et al., 2006; Goodman et al., 2006; Yu et al., 2014), before being endocytosed and recycled back to the Golgi and ER (Belenkaya et al., 2008; Port et al., 2008). Known ERAD components, such as VCP and FAF2, colocalised with EVI/WLS at the ER, indicating that this is where they interfere with EVI/WLS shuttling (Fig. S6B,C). As K63-linked ubiquitylation plays important roles in protein sorting within the endolysosomal compartments (Akutsu et al., 2016; Erpapazoglou et al., 2014; Swatek and Komander, 2016), and thus potentially regulates EVI/WLS recycling, we asked where EVI/WLS would accumulate after knockdown of UBE2N and perturbed K63-linkage formation. For this, we used HeLa cells, which have been previously used for EVI/WLS localisation studies (Gasnereau et al., 2011). As immunofluorescence staining of endogenous EVI/WLS in human cells is difficult, due to limitations of the available antibodies, overexpressed EVI/WLS–V5 was used in combination with siUBE2N. EVI/WLS–V5 accumulated strongly in siUBE2N-transfected cells (Fig. S6D), similar to what was observed for endogenous EVI/WLS in HEK293T cells (Fig. 4). Surprisingly, EVI/WLS–V5 staining remained mostly restricted to the ER after knockdown of UBE2N, possibly indicating that in this system, K63-linked ubiquitylation is important for ER-related degradation rather than endosomal sorting (Fig. S6D).

Accordingly, we envision the following model of how EVI/WLS is ubiquitylated and subjected to ERAD: ERLIN2 serves as an important link between EVI/WLS and other ERAD components and potentially helps to recruit the ubiquitylation machinery, which consists of at least UBE2K, UBE2N, UBE2J2 and CGRRF1. Polyubiquitylated EVI/WLS interacts with FAF2 and UBXN4, which recruit VCP to the ER membrane, resulting in the dislocation and, eventually, the degradation of EVI/WLS (Fig. 6E).

## DISCUSSION

Cellular signalling pathways frequently regulate and are regulated by protein stability. In this study, we investigated the ubiquitylation of the WNT ligand cargo protein EVI/WLS and how it is linked to the ERAD machinery. We performed a small-scale loss-of-function screen using EVI/WLS protein stability as a phenotypic readout (Fig. 1). By this means, we identified five candidates that had increased EVI/WLS protein levels upon knockdown: ERLIN2, FAF2, UBXN4, UBE2K and UBE2N (Figs 1,2 and 4). FAF2 and UBXN4 harbour VCP-interaction domains and are anchored at the ER membrane by an ‘intramembrane’ domain, which leaves both their N- and C-termini facing the cytoplasm (Liang et al., 2006; Meyer and Weihl, 2014; Mueller et al., 2008; Schuberth and Buchberger, 2008). This allows them to maintain a firm grip on VCP and to support it during the generation of mechanical force by ATP-dependent protein extraction from the ER (Hirsch et al., 2009). Our data indicate an interaction between EVI/WLS and ERLIN2 prior to ubiquitylation (Fig. 6A), suggesting a role for ERLIN2 as linker between EVI/WLS and the ERAD machinery, similar to that described for 3-hydroxy-3-methylglutaryl-coenzyme A reductase (HMG-CoA reductase) and inositol 1,4,5-trisphosphate receptor (IP3R) (Jo et al., 2011a,b; Pearce et al., 2007, 2009; Wang et al., 2009). Immunoprecipitation experiments confirmed the interaction of FAF2 and ERLIN2, which has also been reported in a previous study that mapped ERAD component interactions (Christianson et al., 2012). The additional interaction with VCP and PORCN indicates either the formation of a large complex at the ER membrane prior to EVI/WLS degradation or a sequential recruitment of these proteins. Immunofluorescence analysis confirmed the colocalisation of EVI/WLS–V5, GFP-tagged catalytically inactive VCP (VCP-DKO–GFP) and FAF2 at sites of the ER, additionally strengthening the notion that these proteins are involved in the ERAD of EVI/WLS (Fig. S6B,C).

It has been described previously that a complex of ERLIN1 and ERLIN2 is important for the degradation of IP3R (Pearce et al., 2009); however, we found that ERLIN1 did not regulate EVI/WLS protein levels and did not interact with EVI/WLS (Figs 1,3). This is in line with the previously described ERLIN1-independent role of ERLIN2 in the degradation of HMG-CoA reductase (Jo et al., 2011a), indicating several parallel mechanisms involving ERLIN2 that probably depend on additional interaction partners. It is assumed that most ERAD-related proteins have been identified in yeast and mammals (Christianson and Ye, 2014); however, it cannot be excluded that there are additional ERAD-associated proteins that regulate EVI/WLS that could not be detected in our assay due to non-specific siRNAs or cell line dependency.

We found that endogenous EVI/WLS is modified with ubiquitin of several linkage types (K11, K48 and K63; Fig. 6; Fig. S6A), as has been recently described for other ERAD clients (Leto et al., 2019). The presence of K63-linked ubiquitin chains indicates a role in EVI/WLS endocytosis and trafficking as well as degradation. It is well known that EVI/WLS associates with WNT ligands in the ER of WNT-secreting cells and helps to shuttle them to the cell surface (Bänziger et al., 2006; Bartscherer et al., 2006; Goodman et al., 2006; Yu et al., 2014). Afterwards, EVI/WLS is endocytosed with the help of clathrin and recycled back to the Golgi and ER in a retromer-dependent process, where it can bind again to WNTs (Belenkaya et al., 2008; Port et al., 2008). Upon inhibition of EVI/WLS trafficking, EVI/WLS is shuttled to the lysosomes for degradation (Franch-Marro et al., 2008; Gross et al., 2012; Yang et al., 2008). Recently, a study in *Caenorhabditis elegans* found that knockout of UBC-13 (an E2 ubiquitin-conjugating enzyme with K63 specificity; orthologue of human UBE2N) disrupts trafficking of MIG-14 (orthologue of EVI/WLS) and diverts it to lysosomes; however the authors did not show direct ubiquitylation of MIG-14 (Zhang et al., 2018). In agreement with this previous study, we show here that human EVI/WLS is modified with K63-linked ubiquitin by UBE2N and UBE2V2 (Fig. 6C). Surprisingly though, in our study, loss of UBE2N and UBE2V2 led to increased EVI/WLS protein levels and WNT secretion (Fig. 4), and the accumulated EVI/WLS was mostly restricted to the ER (Fig. 6D), indicating that the regulation of EVI/WLS by UBE2N is complex and probably context dependent. In future studies, it will be important to conclusively determine which cues regulate the degradation of EVI/WLS by ERAD versus lysosomal degradation. The apparent reduction in secretion of WNT3 after knockdown of VCP could be due to an effect on viability or because WNT3 might be retained in the secretory machinery so that it cannot be secreted. Notably, knockdown or inhibition of VCP has pleiotropic effects, and different phenotypes, including cell death, can be observed according to the experimental conditions, such as timing and length of the experiments (Glaeser et al., 2018).

We have not yet identified the E3 ubiquitin ligase that is required for UBE2N-mediated ubiquitylation of EVI/WLS, but our data and recently published high-throughput studies indicate that it is presumably not an ER membrane-associated protein, but rather is cytosolic (Fenech et al., 2020; Glaeser et al., 2018; Leto et al., 2019). It should also be considered that previous *in vitro* and structural studies have shown that UBE2N∼ubiquitin together with UBE2V2 can adopt an active conformation even in the absence of an E3 ubiquitin ligase, suggesting E3-independent ubiquitin chain elongation (McKenna et al., 2001; Pruneda et al., 2011). However, sophisticated real-time FRET analysis did not reveal ubiquitin transfer events in the absence of an E3 (Branigan et al., 2020).

Our data indicate that EVI/WLS is additionally ubiquitylated by UBE2K (Fig. 6B), in addition to the previously described UBE2J2 and CGRRF1 (Glaeser et al., 2018). Ubc6, the yeast orthologue of UBE2J2, has been reported to prime substrates with short K11-linked ubiquitin modifications, which are presumably not sufficient to recruit the degradation machinery (Mehrtash and Hochstrasser, 2019; Tsuchiya et al., 2018; Weber et al., 2016; Xu et al., 2009). Therefore, it is tempting to speculate that UBE2K can elongate initial modifications by UBE2J2 or UBE2N in mammalian cells and allow successful interaction with downstream factors, potentially even without an associated E3 ubiquitin ligase (Middleton and Day, 2015; Pluska et al., 2021; Rodrigo-Brenni and Morgan, 2007). It is currently still unclear which amino acids of EVI/WLS are ubiquitylated by which E2 enzyme and E3 protein. Furthermore, Ubc6 can also modify hydroxylated amino acids (Weber et al., 2016), such as serine and threonine. Therefore, further studies are required to characterise the composition and localisation of ubiquitin modifications carried by EVI/WLS.

Our data provides insights into the degradation mechanism of an endogenous substrate of mammalian regulatory ERAD, which seems to function independently of the well-studied E3 ubiquitin ligases HRD1, GP78 and MARCH6 and engages with various E2 ubiquitin-conjugating enzymes. Surprisingly, knockdown of UBE2G2 had no effect on EVI/WLS protein abundance (Fig. 1), although UBE2G2 has recently been reported to be required for the degradation of several other ERAD clients (Leto et al., 2019). In the future, it will be important to test whether other substrates of regulatory ERAD are also independent of UBE2G2 or if this is a cell type- or assay-specific effect.

There are still open questions regarding EVI/WLS degradation, and in particular, the nature of a potential ER membrane channel protein for EVI/WLS dislocation remains elusive, as our screen was not able to identify such a protein. While the extraction of full-length transmembrane proteins from membranes has been described (Fleig et al., 2012; Garza et al., 2009), it is also conceivable that the eight-pass transmembrane protein EVI/WLS is cleaved within the ER membrane and that the parts are extracted separately. It remains questionable whether proteins can be removed from the ER membrane by brute force generated by VCP alone, and a hitherto-undiscovered channel protein or cleaving enzyme seems a more elegant and potentially less energy-intensive approach.

We reported previously that EVI/WLS is a target of regulatory ERAD and that the interaction of EVI/WLS with WNT ligands prevents its degradation. For this previous analysis we used HEK293T cells, a cell line with low endogenous expression of WNTs, to demonstrate the stabilisation of EVI/WLS by overexpressing WNT ligands (Glaeser et al., 2018). Here, we show that this effect can be reversed by inhibiting the lipidation of endogenous WNT ligands in melanoma cells, which naturally produce a lot of endogenous WNT5A (Fig. 5B). Surprisingly, we found that inhibiting the proteasome led to the accumulation of ubiquitylated EVI/WLS even in cells with endogenous WNT ligands, indicating that cells have a surplus production of EVI/WLS, leading to constant turnover in cells with active WNT signalling (Fig. 5D). We cannot conclusively determine at this point which fraction of EVI/WLS is degraded because of misfolding.

Many cancer entities require EVI/WLS and active WNT secretion throughout tumorigenesis (Zhan et al., 2017). Besides WNT signalling, the deregulation of various other cellular communication pathways also leads to cancer development, and many of the underlying mechanisms are closely associated with the UPS (Deng et al., 2020). Thus, it is not surprising that FAF2 and ERLIN2 have also been implicated in cancer, for example in uveal melanoma and breast cancer, respectively (Li et al., 2019; Wang et al., 2012). It will be interesting to test whether the observed phenotypes are connected to EVI/WLS protein abundance and whether WNT signalling and tumour invasiveness could be targeted via ERAD.

## MATERIALS AND METHODS

### Cell lines and culture method

The human melanoma cell line A375 (ATCC CRL-1619) and human embryonic kidney HEK293T cells (ATCC CRL-11268) were purchased from the American Type Culture Collection (ATCC, Manassas, USA). All cells were cultured as monolayers in Dulbecco's Modified Eagle's medium (Gibco DMEM, 41965062; Thermo Fisher Scientific, Waltham, USA) with 4.5 g l^−1^ (high) glucose supplemented with 10% fetal bovine serum (FBS, volume fraction; F7524-500 ML; Sigma-Aldrich, St Louis, USA) without antibiotics at 37°C and 5% CO_2_ in a humidified atmosphere and were regularly confirmed to be mycoplasma negative. The CRISPR/Cas9 *EVI/WLS* knockout (KO) cell lines HEK293T KO2.9 and A375 sgEVI2_4 were generated by Oksana Voloshanenko and Iris Augustin [both Division of Signalling and Functional Genomics, German Cancer Research Center (DKFZ) and Heidelberg University, Germany], respectively, using the guide RNA sgEVI2 (5′-TGGACGTTTCCCTGGCTTAC-3′) and single-cell clonal expansion, according to previously published protocols (Glaeser et al., 2018). All cell lines were authenticated.

### Inhibitor treatment

The porcupine (PORCN) inhibitor LGK974 was used at 10 µM for 96 h before cell lysis with daily medium changes and phosphate-buffered saline (PBS; 10010056; Thermo Fisher Scientific, Waltham, USA) washes (stock solution 50 mM in DMSO; WuXi AppTec, Shanghai, China). The proteasome inhibitor MG132 was used at 1 µM for 24 h (stock solution 10 mM in DMSO; 474791; Merck, Darmstadt, Germany). For all inhibitors, equivalent volumes of DMSO (D8418-50ML; Sigma-Aldrich, St Louis, USA) were used as solvent control.

### siRNA transfection and RNAi experiments

Cells were transfected with siRNAs from Ambion (5 µM stock solution; Thermo Fisher Scientific, Waltham, USA) or Dharmacon (20 µM stock solution; Horizon Discovery Group, Cambridge, UK) using Invitrogen Lipofectamine RNAiMAX Transfection Reagent (13778150; Thermo Fisher Scientific, Waltham, USA) and harvested 72 h later. Details of siRNAs are listed in Table S2.

A total of 7×10^4^ A375 melanoma cells in 2 ml culture medium per well of a 6-well plate were transfected 24 h after seeding after being washed once with PBS. A 3 µl volume of siRNA or 6 µl of RNAiMAX were each mixed with 125 µl of Gibco Roswell Park Memorial Institute (RPMI) 1640 Medium (11875093; Thermo Fisher Scientific, Waltham, USA) and incubated separately at room temperature for 2 min. Then, the two solutions were combined and incubated at room temperature for an additional 5 min before being added to the cells. For TUBE assays, 5×10^5^ A375 cells in 10 ml culture medium were transfected in 10 cm dishes using 20 µl siRNA and 40 µl RNAiMAX in 625 µl RPMI 1640 medium each.

HEK293T cells were reverse transfected with siRNA stock solutions diluted 1:40 in ddH_2_O (working solution). Per well of a 6-well plate, 4 µl RNAiMAX was diluted in 250 µl RPMI 1640 medium and incubated at room temperature for 10 min, then further diluted with 250 µl RPMI 1640 medium. In parallel, 100 µl of siRNA working solution was added to the well and then mixed with 500 µl of the diluted RNAiMAX. A total of 3×10^5^ HEK293T cells were seeded per well in 1.4 ml culture medium after 30 min incubation at room temperature.

### Plasmid generation and transfection

Plasmids for expression of ERLIN1–FLAG (pENTR #18722573, open), ERLIN2–FLAG (pENTR #127630018, open), FAF2–FLAG (pENTR #191683255, open), UBXN4–FLAG (pENTR #178534864, open) and FLAG–UBE2K (pENTR #123919860, open) were generated using the Gateway Cloning system with the destination vectors pDEST-FLAG N-terminal (#1121; for FLAG–UBE2K) and pDEST-FLAG C-terminal (#1124, for all others). A STOP codon was introduced at the end of the coding sequence of FLAG–UBE2K using the Q5 Site-Directed Mutagenesis Kit (E0554S; New England Biolabs, Ipswich, USA), according to the manufacturer's instructions, and the primers 5′-TGATTGGACCCAGCTTTCTTG-3′ and 5′-GTTACTCAGAAGCAATTCTG-3′.

The plasmid encoding PORCN–FLAG was obtained from OriGene Technologies, Rockville, USA (pCMV6-Myc-DDK-tagged PORCN, #RC223764). pRK5-HA-ubiquitin constructs (Addgene 17608, 17606 and 17605, Lim et al., 2005; Addgene 22901, Livingston et al., 2009), pLX302 Luciferase-V5 puro (Addgene 47553, Kang et al., 2013), VCP(DKO)-EGFP (Addgene 23974, Tresse et al., 2010) and pcDNA Wnt3 (Addgene 35909, Najdi et al., 2012) were obtained from Addgene, Watertown, USA. The pcDNA-NanoLuc-Wnt3 plasmid was generated by introducing the NanoLuciferase (Hall et al., 2012) sequence after amino acid position W26 of the pcDNA Wnt3 plasmid. pcDNA-V5-hWls K410/419R (EVI/WLS–V5 K410/419R) was derived from pcDNA-V5-hWls (EVI/WLS–V5, Belenkaya et al., 2008) using the QuikChange II Site-Directed Mutagenesis Kit (200523; Agilent Technologies, Santa Clara, USA), according to the manufacturer's instructions, and the primers 5′-CGGAACATCAGTGGGAGGCAGTCCAGCCTGCCAGCTATGAGCAGAGTCCGGCGGC-3′ and 5′-GCCGCCGGACTCTGCTCATAGCTGGCAGGCTGGACTGCCTCCCACTGATGTTCCG-3′.

For plasmid transfection, 5×10^5^ A375 cells or 3.5×10^6^ HEK293T cells were seeded in 10 ml culture medium in 10 cm dishes the day before the transfection. The next day, 1.5 µg plasmid DNA was diluted in 500 µl serum-free RPMI 1640 and supplemented with 12 µl TransIT-LT1 Transfection Reagent (731-0027; Mirus Bio, Madison, USA). After 15 min incubation at room temperature, the mixture was added dropwise to the cells. Cells were harvested 48 h after transfection. For one well of a 6-well plate, 7×10^4^ A375 cells or 2×10^5^ HEK293T cells were seeded in 2 ml culture medium and transfected with 1 µg plasmid DNA in 250 µl RPMI 1640 medium and 5 µl transfection reagent (unless indicated otherwise).

### RT-qPCR

For mRNA expression analysis, total RNA was isolated from cultured cells using the RNeasy Mini Kit (74106; QIAGEN, Hilden, Germany) with on-column DNase digestion using the RNase-Free DNase Set (79254; QIAGEN, Hilden, Germany), both according to the manufacturer's instructions (quick start protocol version March 2016, including optional centrifugation step at full speed). cDNA synthesis was performed in 1.5 ml tubes using the RevertAid H Minus First Strand cDNA Synthesis Kit (K1632; Thermo Fisher Scientific, Waltham, USA) with 1–5 µg of total RNA input and oligo (dT)_18_ primers, following the manufacturer's instructions, before being diluted to 5–10 ng/µl with ddH_2_O. mRNA expression was quantified in technical triplicates using RT-qPCR performed in 384-well plates on a Roche LightCycler 480 Instrument II with dual hybridisation probes from The Universal ProbeLibrary (Roche, Basel, Switzerland). Oligonucleotide sequences used for RT-qPCR are listed in Table S3. *GAPDH* and *SDHA*, *G6PD* or *ACTB* served as reference genes, and relative mRNA expression levels were calculated using the Pfaffl method with siControl, siLuciferase or DMSO treatment as calibrators. Mean and confidence intervals were calculated using R (R Version 3.6.1, R Studio version 1.2.1335).

### Blue Sepharose assay

Secreted WNTs were enriched from cell culture supernatants using the affinity chromatography resin Blue Sepharose 6 Fast Flow (17-0948-01; GE Healthcare, Chicago, USA) and analysed by western blotting according to previously published protocols (Glaeser et al., 2016). In brief, 2 ml cell culture medium from a well of a 6-well plate of nearly confluent cells was collected 24 h after medium change and centrifuged at room temperature for 10 min at 8000 ***g***. The supernatant was then transferred to a new tube, and Triton X-100 (T8787-250 ml; Sigma-Aldrich, St Louis, USA) was added to reach a final volume fraction of 1%. Per sample, 30 µl of Blue Sepharose 6 Fast Flow resin was washed twice in washing buffer [50 mM Tris-HCl (75746-250G, Sigma-Aldrich, St Louis, USA), pH 7.5, 150 mM KCl (P-9541, Sigma-Aldrich, St Louis, USA) and a volume fraction of 1% Triton X-100 in ddH_2_O] by centrifugation and decanting of supernatant (3 min, 2800 ***g***). Then, resin was distributed equally to all samples and incubated overnight at 4°C in a tube rotator. The following day, resin was washed two or three times, as above, until the wash buffer was clear. After the last wash, resin was taken up in 200 µl 1× sodium dodecyl sulfate (SDS; 75746-250G; Sigma-Aldrich, St Louis, USA) buffer. The samples were boiled at 95°C for 5 min, and 40 µl of the sample used for SDS polyacrylamide gel electrophoresis (SDS–PAGE).

### Immunoprecipitation

To investigate protein interactions within the ERAD pathway, FLAG-tagged proteins were overexpressed in wild-type and *EVI/WLS* KO HEK393T cells in 10 cm dishes, and protein lysates were harvested 48 h later in 600 µl eukaryotic lysis buffer [20 mM Tris-HCl, pH 7.4, 130 mM NaCl (31434-M, Sigma-Aldrich, St Louis, USA), 2 mM ethylenediaminetetraacetic acid (EDTA; sc-204735; Santa Cruz Biotechnology, Dallas, USA) and glycerol at a volume fraction of 10% (G5516; Sigma-Aldrich, St Louis, USA), supplemented with protease inhibitor (11836153001; Sigma-Aldrich, St Louis, USA) and Triton X-100 at a volume fraction of 1%]. To investigate EVI/WLS ubiquitylation, pRK5-HA-ubiquitin constructs (wild-type, K11, K48 and K63 ubiquitin) were overexpressed in wild-type and *EVI/WLS* KO A375 cells in 10 cm dishes, and protein lysates were harvested 72 h later in eukaryotic lysis buffer. The HA-tagged K11, K48 and K63 ubiquitin mutants can only make linkages of the indicated types. For endogenous immunoprecipitations (IPs), wild-type and *EVI/WLS* KO HEK393T cells were harvested in lysis buffer containing 50 mM Tris-HCl, pH 7.5, 150 mM NaCl, 1 mM EDTA, glycerol at a volume fraction of 10% and 0.25% sodiumdeoxycholate (mass fraction; D6750-10G; Sigma Aldrich, St. Louis, USA), which was supplemented with protease inhibitor and NP-40 alternative (492016-100ML; Merck, Darmstadt, Germany) at a volume fraction of 1% before use.

After cell harvesting, complete cell lysis was ensured by incubation on a tube rotator at 4°C for 30 min, and protein lysates were clarified by centrifugation at full speed for 20 min in a table-top centrifuge at 4°C. Protein content was quantified using a Pierce bicinchoninic acid (BCA) Protein Assay kit (23225; Thermo Fisher Scientific, Waltham, USA) and a Mithras LB 940 multimode microplate reader (Berthold Technologies, Bad Wildbad, Germany). Equal amounts of protein (1–3.5 mg) were used for pulldowns. Per sample, 40 µl anti-FLAG M2 Affinity Gel (A2220; Merck, Darmstadt, Germany) or 15 µl monoclonal anti-HA agarose (A2095; Merck, Darmstadt, Germany) and equal amounts of control agarose beads (negative control; UM400; LifeSensors, Malvern, USA) were washed twice with 750 µl lysis buffer without Triton X-100 (centrifugation in between for 30 s at 5000 ***g***), then blocked for 1 h with 2.5% bovine serum albumin (BSA; mass fraction; 1501-0500; GERBU Biotechnik, Heidelberg, Germany) in Tris-buffered saline with Tween-20 [TBST; 10× TBST contains 1.37 M NaCl, 200 mM Tris-HCl, pH 7.6 and 1% volume fraction of Tween-20 (P9416; Sigma-Aldrich, St. Louis, USA)] at 4°C on a tube rotator and washed again twice as previously. The resin was then equally distributed to all samples and incubated overnight at 4°C in a tube rotator. For endogenous IPs, 1 mg protein per condition was mixed with 1 µl of antibodies (see Table S4 for details) and 50 µl of magnetic Dynabeads Protein G slurry (10004D; Life Technologies, Carlsbad, USA) before overnight incubation at 4°C. Magnetic beads were washed and blocked as described above for resin.

The next day, the resin or beads were washed four to seven times, as described above. Proteins with FLAG- or HA-tag and their interacting proteins were eluted using 100 µl of 150 ng µl^−1^ 3× FLAG Peptide (F4799; Merck, Darmstadt, Germany) or HA peptide (HY-P0239; Hölzel Diagnostika, Cologne, Germany) and incubation for 30 min at 4°C in a tube rotator, according to the manufacturer's instructions. Then, samples were centrifuged as described above, and 100 µl of the supernatant was transferred to a new tube. Pulldown samples or input controls (∼15 µg protein of the original clarified lysates) were prepared for SDS–PAGE by combining them with 1/5 volume fraction of 5× SDS buffer [312.5 mM Tris-HCl, pH 6.8, 0.5 M dithiothreitol (DTT; A2948,0005; AppliChem, Darmstadt, Germany), 10% (mass fraction) SDS, 0.1% (mass fraction) Bromophenol Blue (B5525-25G; Sigma-Aldrich, St Louis, USA) and a volume fraction of 10% Tris-(2-carboxyethyl)-phosphin (TCEP; 77720; Thermo Fisher Scientific, Waltham, USA) and 50% glycerol] and 5 min incubation at 95°C. For endogenous IPs, washing buffer was removed completely from the magnetic beads using a magnetic rack, beads were mixed with 100 µl 1× SDS buffer and then incubated at 95°C for 5 min.

### Tandem ubiquitin-binding entity assays

Ubiquitylated proteins were analysed using TUBE2 (agarose, 30 µl per sample; UM402; LifeSensors, Malvern, USA), TUBE1 (magnetic beads, 15 µl per sample; UM401M; LifeSensors, Malvern, USA), or K48 and K63 TUBE (FLAG; UM607 and UM604, respectively; LifeSensors, Malvern, USA), according to the manufacturer's instructions, with control agarose beads as negative control. For buffer compositions, the protein isolation protocol and elution using 3× FLAG peptide see the ‘Immunoprecipitation’ section above. Pulldowns were done from one 10 cm dish per condition, with 5×10^5^ A375 cells seeded 1 day before the start of drug treatment or siRNA/plasmid transfection. Cell harvesting was done in eukaryotic lysis buffer with Triton X-100 at a volume fraction of 1%, protease inhibitor, 5 mM N-ethylmaleimide (E3876-5G; Sigma-Aldrich, St Louis, USA) and 2 mM 1,10-phenanthroline (P9375-1G; Sigma-Aldrich, St Louis, USA), as well as 250 nM FLAG K48 or K63 TUBE if applicable, and was followed by protein quantification. Then, all samples were adjusted to contain the same amount of protein (0.5–1 mg), diluted with lysis buffer containing inhibitors to adjust the Triton X-100 concentration to 0.1%, and incubated with TUBEs overnight on a tube rotator at 4°C (TUBE1 and TUBE2) or pre-incubated with 250 nM FLAG K48 or K63 TUBE for 2 h before overnight incubation with 15 µl anti-FLAG M2 Affinity Gel or control agarose beads at 4°C on a tube rotator (FLAG K48 or K63 TUBE). The next day, samples were washed 4× in eukaryotic lysis buffer without Triton X-100 but with inhibitors, and proteins were eluted using 3× FLAG peptide (FLAG K48 or K63 TUBE) or by taking up beads in 100 µl 1× SDS buffer and boiling at 95°C for 5 min (TUBE1 and TUBE2). For input controls, 40 µg of the original protein lysates was diluted to 200 µl with ddH_2_O and prepared for SDS–PAGE with 50 µl 5× SDS buffer by boiling for 5 min at 95°C. Of this, ∼ 6.5 µg were used for western blotting.

### SDS–PAGE and western blotting

Total cellular protein lysates were isolated using eukaryotic lysis buffer (IPs and TUBE assays) or 8 M urea in PBS (all other assays; A1049,1000; AppliChem, Darmstadt, Germany) and prepared for SDS–PAGE as described in ‘Immunoprecipitation’ section above. 15–30 µg of protein lysate was used per sample harvested in 8 M urea/PBS. SDS–PAGE was performed using Invitrogen Bolt 4–12% Bis-Tris Plus Gels (NW04120BOX, NW04122BOX or NW04125BOX; Thermo Fisher Scientific, Waltham, USA) in 1× running buffer with 3-(N-morpholino)propanesulfonic acid [MOPS; 20× running buffer: 1 M MOPS (A1076; AppliChem, Darmstadt, Germany), 1 M Tris-base, 20 mM EDTA and 69.3 mM SDS in ddH_2_O] and with the PageRuler Prestained Protein Ladder (26617; Thermo Fisher Scientific, Waltham, USA). Proteins were transferred to Amersham Protran NC nitrocellulose membranes (10600002; Cytiva, Marlborough, USA) by wet blotting in 1× transfer buffer [20× transfer buffer: 500 mM Bicine (sc-216087A; Santa Cruz Biotechnology, Dallas, USA), 500 mM Bis-Tris (sc-216088A; Santa Cruz Biotechnology, Dallas, USA) and 20 mM EDTA] with 10% methanol (32213; Sigma-Aldrich, St Louis, USA). Membranes were blocked in TBST containing 5% (mass fraction) skim milk (70166-500G; Sigma-Aldrich, St Louis, USA) for 30 min at room temperature and then incubated with primary antibodies overnight at 4°C. All antibodies and dilutions are listed in Table S4. The next day, membranes were washed three times for 7 min in TBST on a shaker and incubated with horseradish peroxidase (HRP)-coupled secondary antibodies for 1 h at room temperature (see Table S4 for details of antibodies and dilutions) and again washed as before. Then, membranes were incubated with enhanced chemiluminescence (ECL) substrates, and the HRP-induced light signals were captured using Amersham Hyperfilm ECL (GE28-9068-36; Cytiva, Marlborough, USA) and made visible using a COMPACT 2 NDT (PROTEC, Oberstenfeld, Germany) developing machine. Immobilon western HRP substrate (WBKLS0100; Merck, Darmstadt, Germany) was used for standard applications, and SuperSignal West Femto maximum sensitivity substrate (34095; Thermo Fisher Scientific, Waltham, USA) was used if stronger signal amplification was necessary. Biological replicates were excluded from the analysis if either siRNA or plasmid transfection did not work.

### NanoLuciferase–WNT3 secretion assay

Luciferase assays were performed in a 384-well format using white, flat-bottom polystyrene plates (781073; Greiner Bio-One, Frickenhausen, Germany) with at least seven technical replicates per biological replicate. On day 1, ∼2500 HEK293T cells in 50 µl culture medium were reverse transfected using 5 µl of 0.2 µM (Dharmacon) or 0.05 µM (Ambion) siRNA and 0.1 µl RNAiMAX in 10 µl serum-free RPMI 1640 medium per well. The next day, cells were additionally transfected using 0.1 µl TranIT-LT1 with 1 ng NanoLuciferase–WNT3 and 5 ng firefly luciferase for normalisation in 10 µl serum-free RPMI 1640 medium per well. 48 h later, the plate was centrifuged for 2 min at 650 ***g***, and 20 µl of the medium was transferred to a second plate to measure NanoLuciferase–WNT3 in the supernatant. NanoLuciferase–WNT3 activity in supernatant and cell lysates, was detected using the Promega Nano-Glo (N1130; Fitchburg, USA) system and a Mithras LB 940 Multimode Microplate Reader (Berthold Technologies; Bad Wildbad, Germany). Luminescence signals in the supernatant were normalised to NanoLuciferase signals and firefly luciferase signals in the cell lysates.

### Microscopy, immunofluorescence staining, imaging and image analysis

All steps were carried out at room temperature unless stated otherwise, and samples were protected from light after addition of fluorescent dyes. HeLa cells were seeded on cover glasses (if applicable, HeLa cells were transfected with siRNAs as described above and seeded on cover glasses 24 h later). The next day, cells were additionally transfected with constructs encoding EVI/WLS–V5 and VCP-DKO–GFP and fixed 24 h later using 4% (volume fraction) paraformaldehyde (A3813,1000; AppliChem, Darmstadt, Germany) in PBS for 10 min at room temperature, followed by three washes with PBS for 5 min. Then, plasma membranes were permeabilised using 0.2% Triton X-100 in PBS (volume fraction) for 10 min and blocking solution [1% goat serum (5425S; Cell Signaling Technology, Danvers, USA), 3% FBS and 0.1% Triton X-100, all volume fraction in PBS] was added for at least 30 min. Primary antibodies diluted in 200 μl PBS (for 24-well plate wells) were added for 1 h at room temperature or overnight at 4°C (for dilutions refer to Table S4), followed by three 5 min washes with PBS on a shaker at high speed. Secondary antibodies were diluted (Table S4) and added for 1 h, followed by three washes as above. Ultimately, cover glasses were inverted and mounted on microscope slides using ProLong Diamond Antifade mountant with 4′,6-diamidino-2-phenylindole (DAPI; P36962; Thermo Fisher Scientific, Waltham, USA). Images were acquired in the .czi format using a Zeiss motorised inverted Axio Observer.Z1 microscope (Cell Observer; Carl Zeiss, Oberkochen, Germany) with the ZEISS ZEN (blue edition) software provided by the DKFZ Light Microscopy Facility (excitation sources: mercury arc burner HXP 120 and LED module Colibri; detector: greyscale CCD camera AxioCam; filter sets: 49(DAPI), 38 HE (eGFP), 43 HE (Cy3) and 50 (Cy5); objective: 63×/1.4 Oil Pln Apo DICIII), and brightness and contrast were adjusted using Fiji (Version 1.51).

Corrected total cell fluorescence (CTCF) was calculated using Fiji (Version 1.51) after selecting single cells using the freehand selection tool and measuring area and mean grey value within the selection. Background fluorescence was determined after selecting regions without cells. The average fluorescence of the background readings multiplied by the area of the selected cell was then subtracted from the cell's integrated density (product of the area and mean grey value) to calculate the CTCF.

### Proliferation assays

Time-lapse live-cell imaging to assess proliferation capacities of A375 melanoma cells were performed using an IncuCyte ZOOM system (40239; Essen BioScience, Ann Arbor, USA) together with the IncuCyte Basic Software (2013B Rev1; Essen BioScience, Ann Arbor, USA). Cells were transfected with siRNA as described above. The next day, 2000 cells per well were seeded in 300 μl of culture medium in transparent, flat-bottom 96-well plates as five technical replicates and were imaged every 2 h using a 10× objective. Confluence of cells was quantified from four images per well.

### Statistical analysis

The non-parametric one-sample or two-sample Wilcoxon tests for data without normal distribution were conducted in R (alternative=‘two-sided’) to assess statistical significance where indicated (R Version 3.6.1, R Studio version 1.2.1335). At least six biological replicates were analysed to ensure adequate power to detect effects. Single data points from independent experiments are shown together with mean and confidence intervals.

## Supplementary Material

Supplementary information

Reviewer comments
