# Supplementary material for: EVI/WLS function is regulated by ubiquitylation and is linked to ER-associated degradation by ERLIN2
Source: J Cell Sci. 2021 Aug 18;134(16):jcs257790. doi: 10.1242/jcs.257790 (PMC8435288; doi:10.1242/jcs.257790)
Supplement: Supplementary information [file joces-134-257790-s1.pdf]

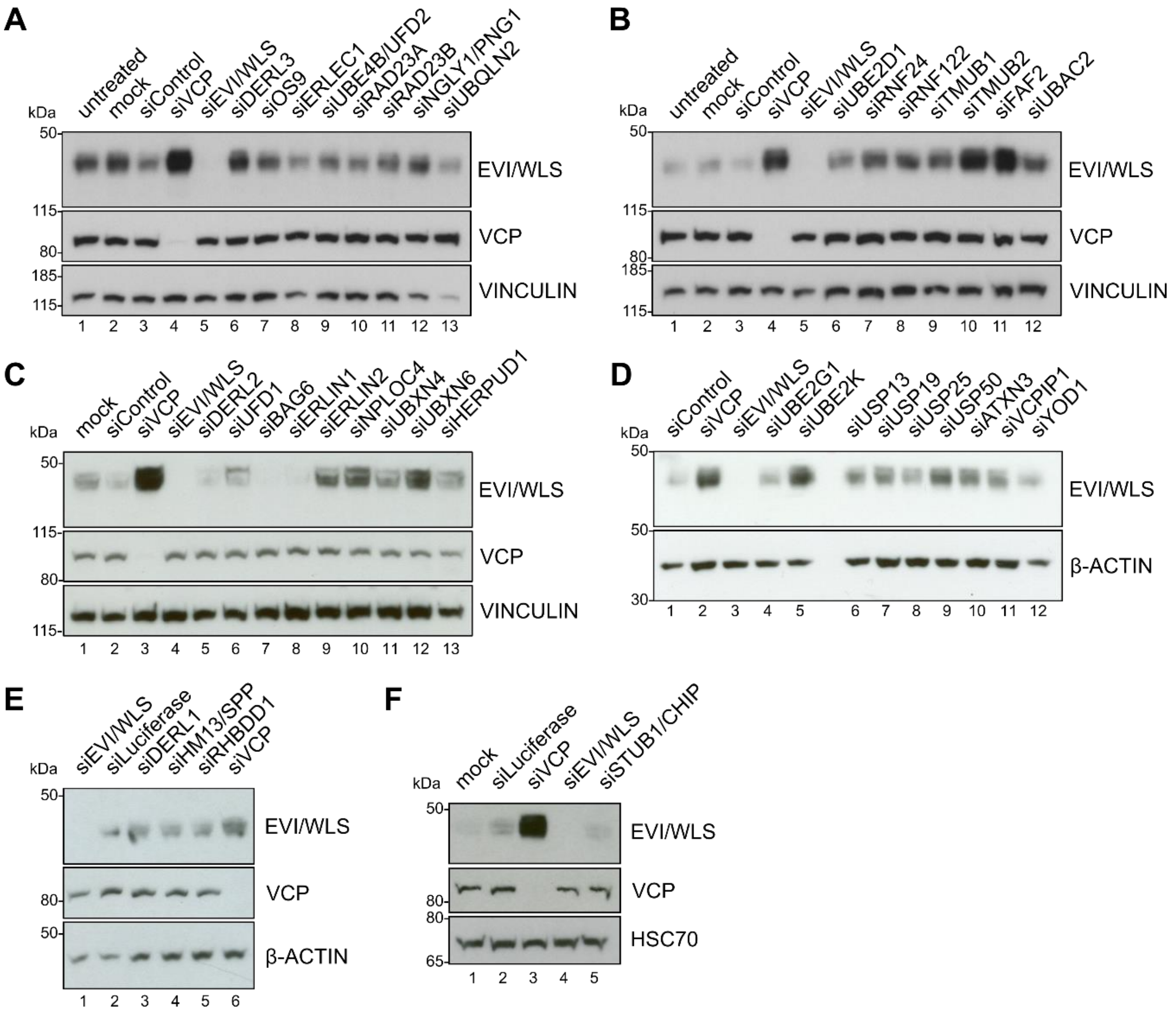

Fig. S1. siRNA-based low-throughput screen identifies novel candidates involved in the degradation of EVI/WLS in HEK293T cells

EVI/WLS protein levels were analysed after siRNA mediated knock-down of target genes. Increased EVI/WLS protein levels compared to siControl/siLuciferase treatment indicated the candidate's possible involvement in EVI/WLS's ERAD. HEK293T cells were harvested 72 h after transfection of the indicated siRNAs. VINCULIN, HSC70, or β-ACTIN served as loading controls. Western blots are representative of three independent experiments. kDa = kilodalton

Lanes 1-5 of **D** are also shown in Figure 1 and were not cut here to maintain integrity of the blot.

Journal of Cell Science • Supplementary information

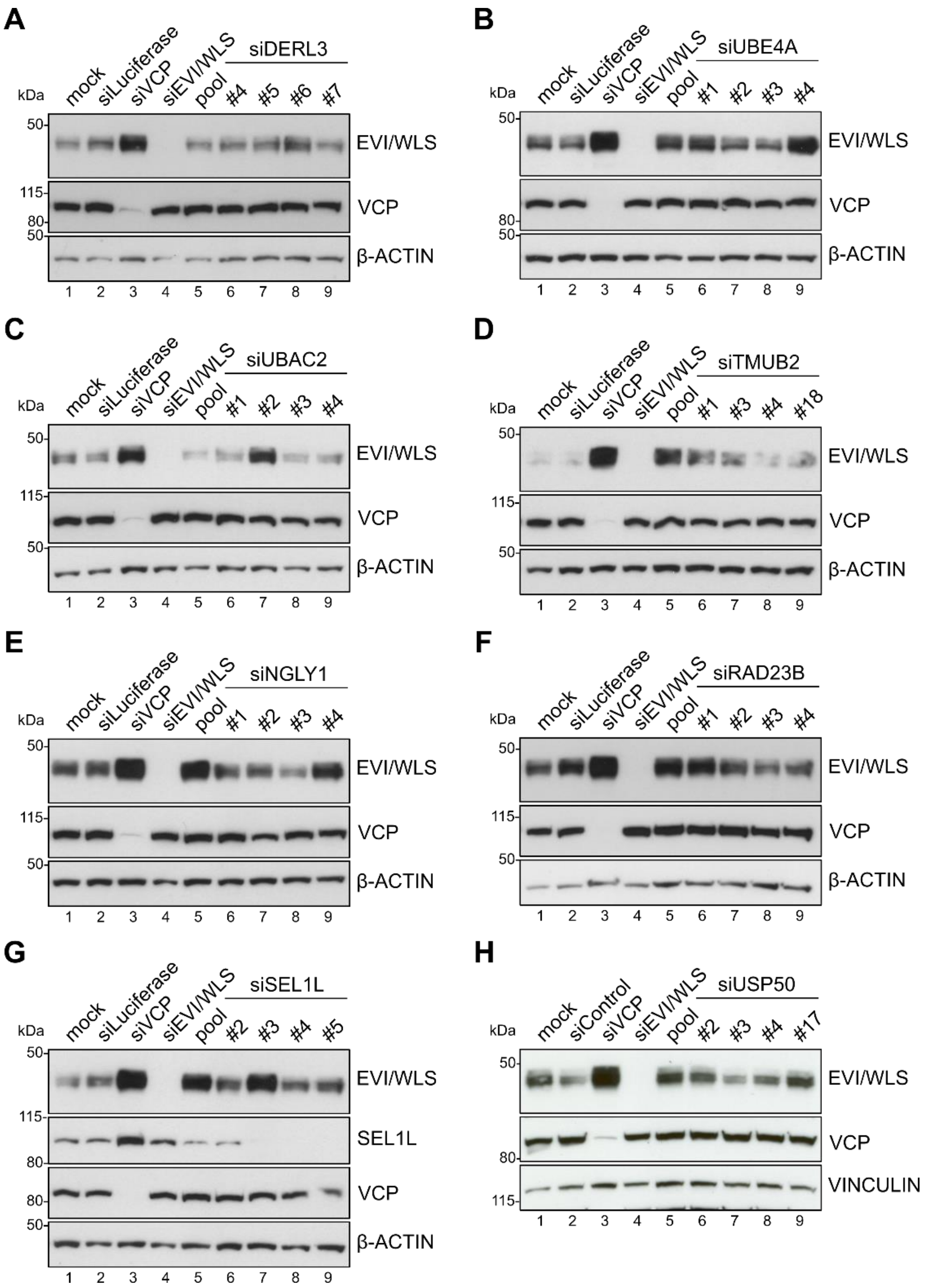

Fig. S2. The knock-down of DERL3, UBE4A, UBAC2, TMUB2, NGLY1, RAD23B, SEL1L, or USP50 by single siRNAs did not show consistent upregulation of EVI/WLS

EVI/WLS protein levels were not markedly elevated or varied between biological replicates after transfection of single or pooled siRNAs against the investigated candidates. HEK293T cells were harvested 72 h after transfection of the indicated siRNAs. Each gene's mRNA was targeted by either single siRNAs or an equimolecular mix of all four respective siRNAs (pool) to analyse their effect on EVI/WLS protein level. VINCULIN or β-ACTIN served as loading control. Western blots are chosen from three independent experiments. kDa = kilodalton

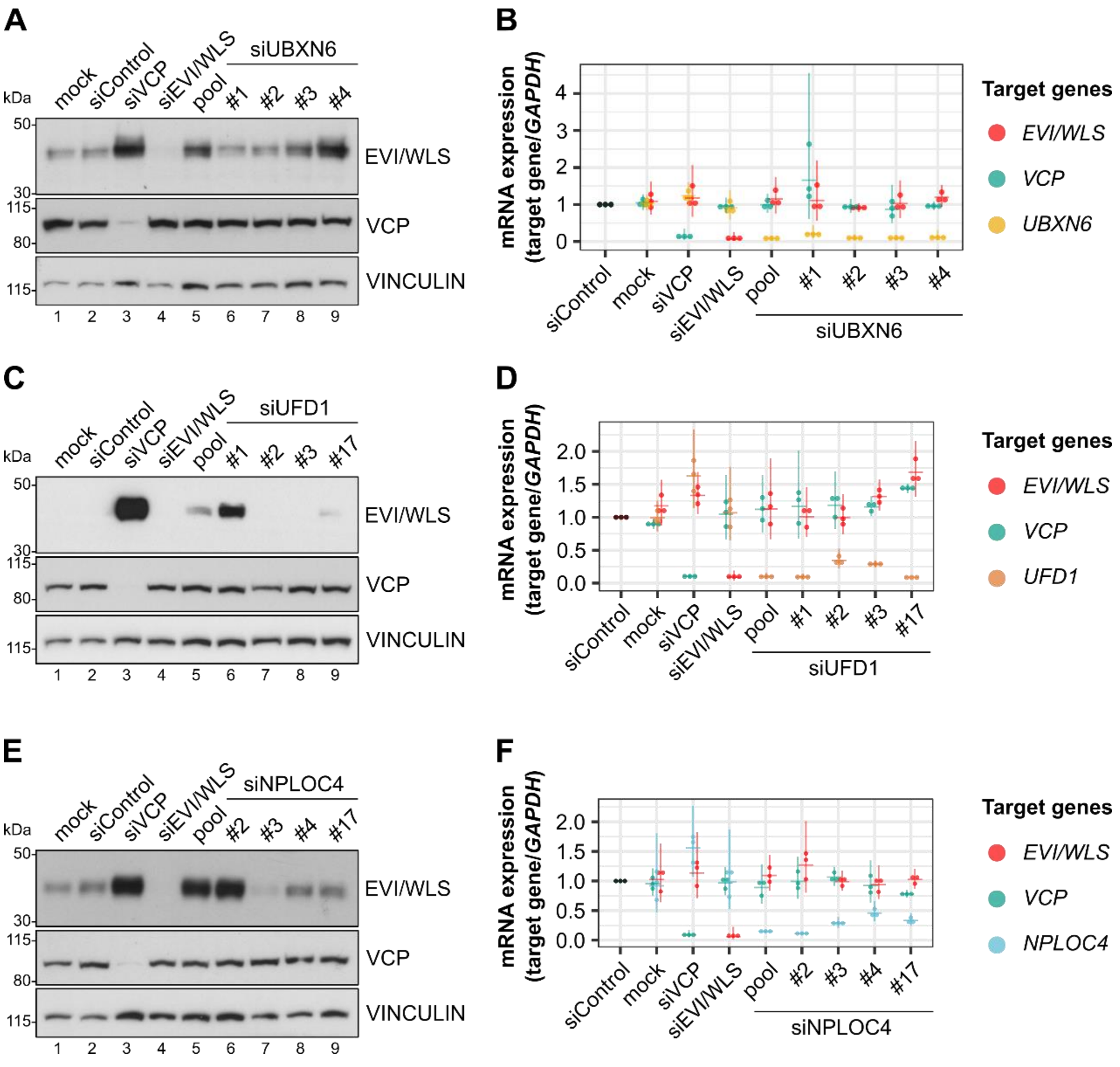

Fig. S3. The knock-down of UBXN6, UFD1, or NPLOC4 by single siRNAs did not show consistent upregulation of EVI/WLS

EVI/WLS protein levels were not markedly elevated or varied between biological replicates after transfection of single or pooled siRNAs against the investigated candidates (**A, C, E**). mRNA expression analyses demonstrated mostly efficient gene silencing by pooled or single siRNAs with little effects on other investigated mRNAs (**B, D, F**).

HEK293T cells were harvested 72 h after transfection of the indicated siRNAs. Each gene's mRNA was targeted by either single siRNAs or an equimolecular mix of all four respective siRNAs (pool) to analyse their effect on EVI/WLS protein level or mRNA expression. **A, C, E.** Total cell lysates were analysed by SDS-PAGE and Western blotting for the specified proteins. VINCULIN served as loading control. Western blots are representative of three independent experiments. kDa = kilodalton

B, D, F. Total cellular RNA was transcribed to cDNA and used for mRNA expression analyses by RT-qPCR. Target gene expression was normalised to siControl treatment and *GAPDH* served as reference gene. Individual data points from three independent experiments are shown with mean and confidence intervals.

Journal of Cell Science • Supplementary information

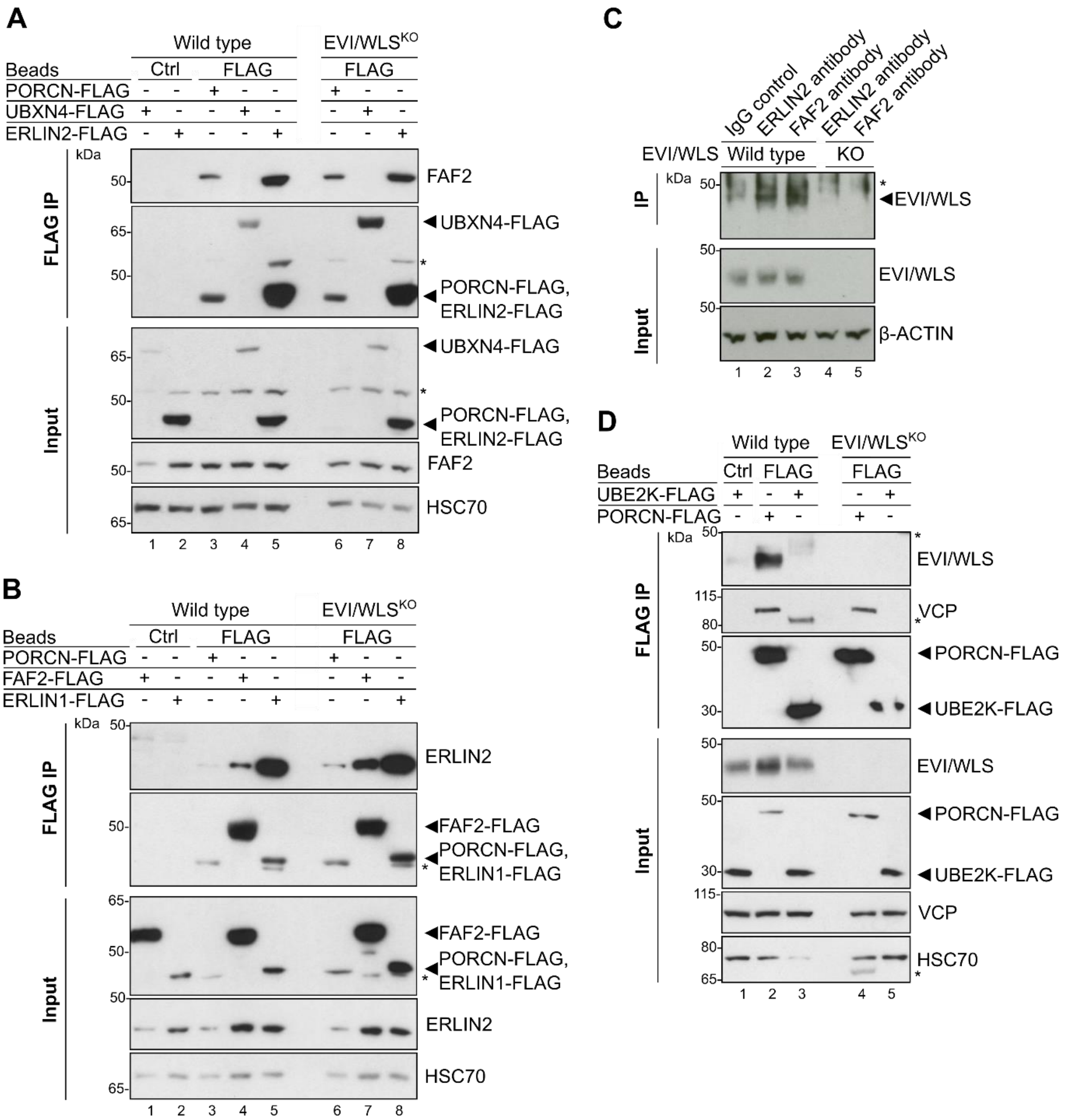

Fig. S4. FAF2 and ERLIN2 interact with each other, but UBE2K and EVI/WLS do not

A, B. Immunoprecipitation (IP) experiments confirmed that endogenous FAF2 and ERLIN2 interact with PORCN-FLAG and each other. Furthermore, endogenous ERLIN2 interacts with ERLIN1-FLAG. HEK293T wild type and EVI/WLS knock-out (EVI/WLS^{KO}) cells were transfected with UBXN4-FLAG, ERLIN1-FLAG, ERLIN2-FLAG, FAF2-FLAG, or PORCN-FLAG overexpression plasmids. After 48 h, total cell lysates were sampled for input control or used for FLAG IP to precipitate FLAG-tagged proteins and their interaction partners. Asterisks mark signal from previous stainings. Results are representative of three independent experiments.

C. IP experiments confirmed that endogenous ERLIN2 and FAF2 interact with endogenous EVI/WLS. HEK293T wild type and EVI/WLS knock-out (EVI/WLS^{KO}) cells were harvested and total cell lysates were sampled for input control or used for IP using antibodies to precipitate ERLIN2 or FAF2 proteins and their interaction partners. Asterisks mark signal from immunoglobulin heavy chains. Results are representative of three independent experiments.

D. The binding of endogenous EVI/WLS to UBE2K-FLAG could not be detected by IP. HEK293T wild type and EVI/WLS knock-out (EVI/WLS^{KO}) cells were transfected with UBE2K-N-FLAG or PORCN-FLAG overexpression plasmids. After 48 h, total cell lysates were sampled for input control or used for FLAG IP to precipitate FLAG-tagged proteins and their interaction partners. Asterisks mark nonspecific signals. Results are representative of two independent experiments.

HSC70 or β-ACTIN served as loading control. kDa = kilodalton

Journal of Cell Science • Supplementary information

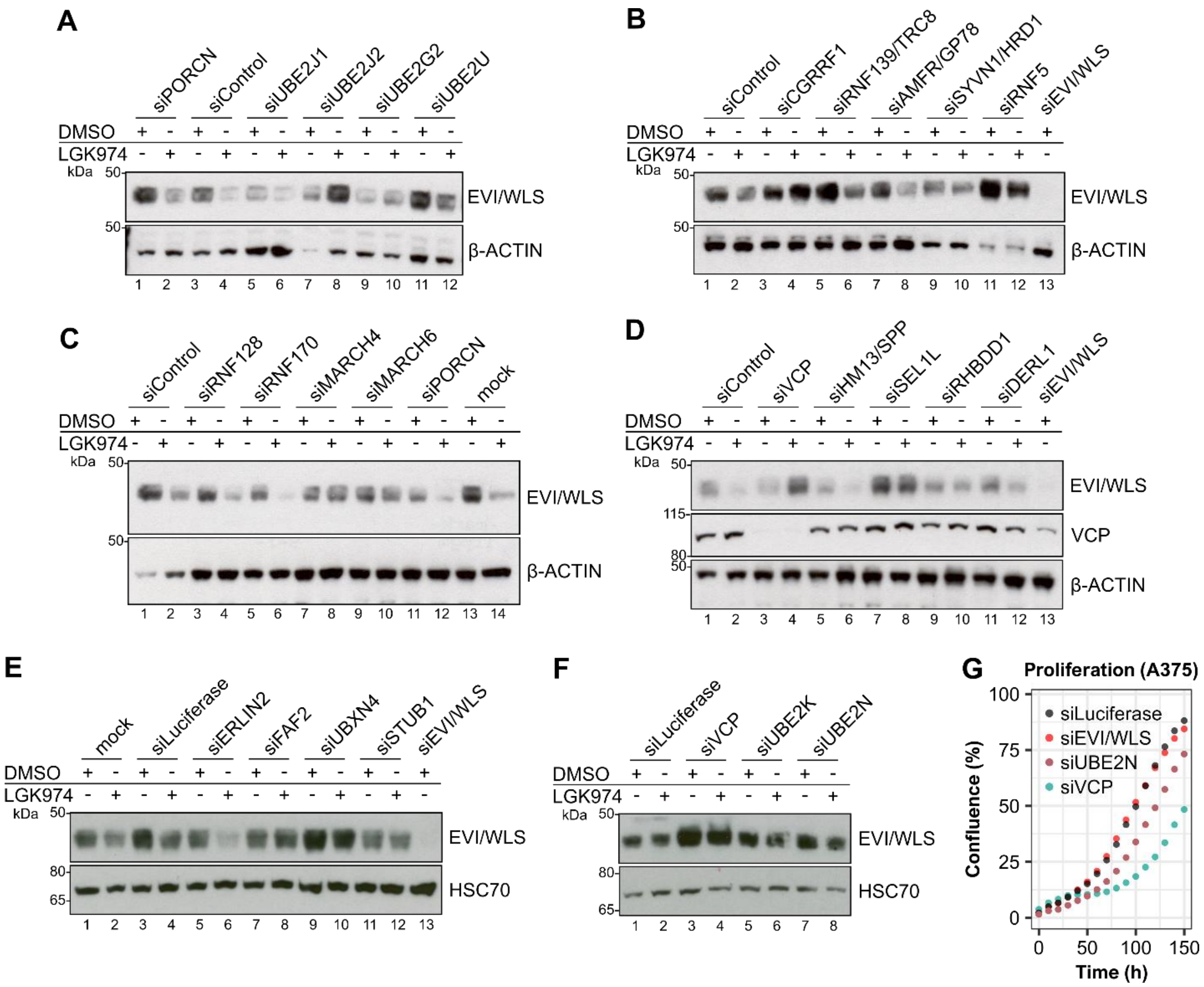

Fig. S5. EVI/WLS is degraded with the help of VCP, CGRRF1, and UBE2J2 in A375 melanoma cells

A, B, C, D, E, F. Knock-down of VCP, CGRRF1, or UBE2J2 prevented degradation of EVI/WLS after LGK974 treatment. A375 melanoma cells were treated with LGK974 (10 µM) with daily medium changes. 24 h after addition of LGK974 cells were transfected with the indicated siRNAs. Again 72 h later, total cell lysates were analysed by SDS-PAGE and Western blotting for the specified proteins. β-ACTIN or HSC70 served as loading control. Western blots are representative of three independent experiments. kDa = kilodalton

G. A375 melanoma cell proliferation is reduced after transfection of siUBE2N or siVCP. Starting from 24 h after transfection of indicated siRNAs, proliferation was analysed by time-lapse live-cell imaging with the IncuCyte ZOOM system together with the IncuCyte Basic Software to determine total confluence of cells. Plot is representative of three independent experiments.

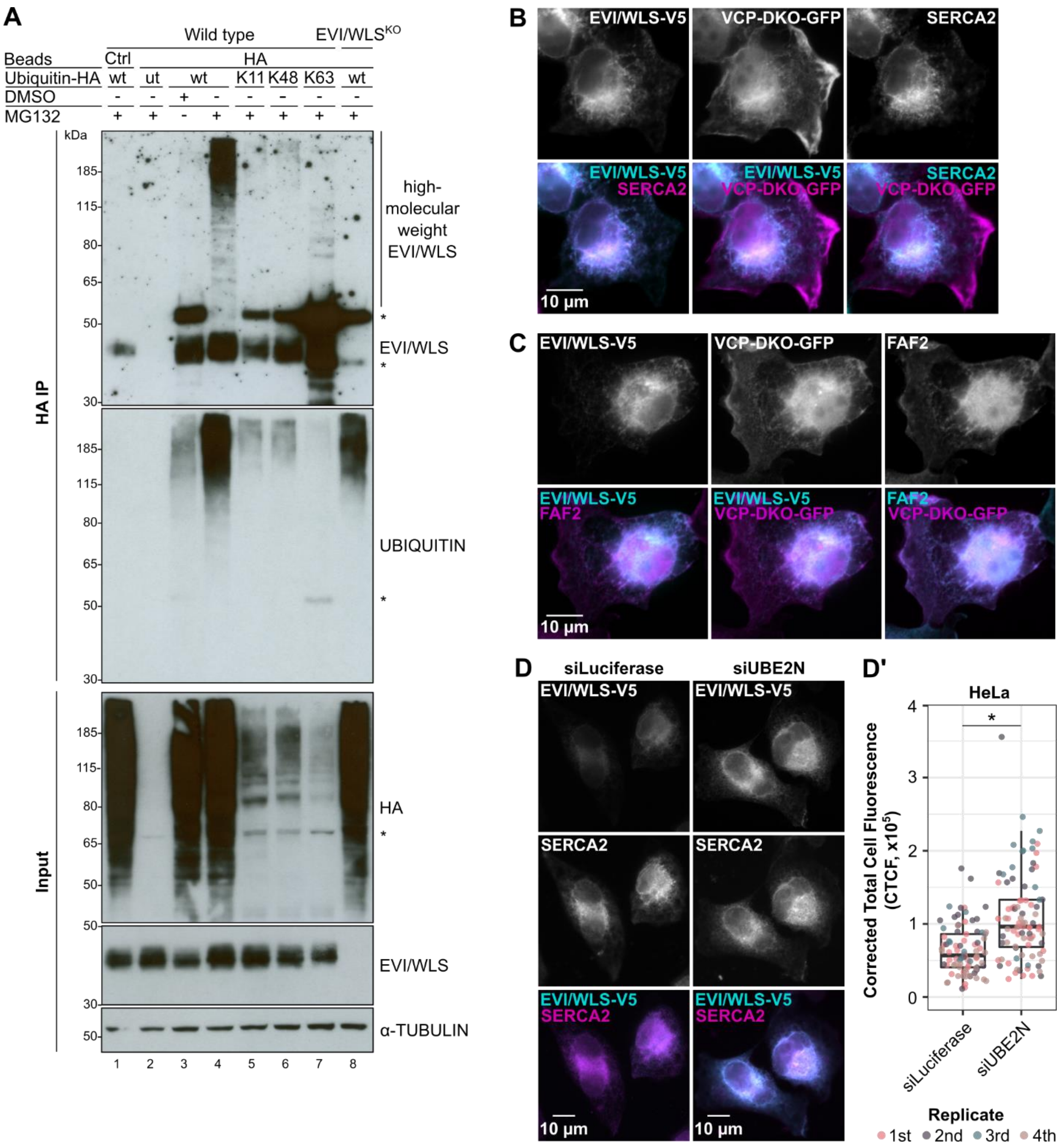

Fig. S6. EVI/WLS is modified with K11-, K48-, and K68-linked ubiquitin and colocalises with VCP and FAF2 in the endoplasmic reticulum (ER)

A. Endogenous EVI/WLS is modified with ubiquitin linked via K11, K48, and K63. A375 wild type (wt) and EVI/WLS knock-out (EVI/WLS^{KO}) cells were transfected with pRK5-HA-Ubiquitin wt, K11, K48, or K63 overexpression plasmids or left untreated (ut). The K11, K48, and K63 ubiquitin constructs can only be elongated with further ubiquitins at the specified position, all others have been mutated to arginines. 24 h before harvest, samples were treated with the proteasome inhibitor MG132 (1 µM) or equivalent volume of DMSO as solvent control. After 72 h, total cell lysates were sampled for input control or used for HA immunoprecipitation to analyse proteins modified with HA-tagged ubiquitin. α-TUBULIN served as loading control. Asterisks mark nonspecific signals. Western blots are representative of three independent experiments. kDa = kilodalton

B, C. EVI/WLS-V5 colocalises with VCP-DKO-EGFP and FAF2 in the ER. HeLa cells seeded on cover glasses were transfected with EVI/WLS-V5 and a catalytically dead mutant form of VCP-EGFP (VCP-DKO-GFP) the next day. Again 24 h later, cells were fixed with 4% paraformaldehyde/PBS and stained for V5 (EVI/WLS) and FAF2 or SERCA2 as ER marker. Images were acquired using a Zeiss motorised inverted Observer.Z1 microscope with the ZEISS ZEN (blue edition) software and processed using Fiji (Fiji is just ImageJ). Image brightness and contrast were adjusted. Shown are representative images of three independent experiments. Scale bar is 10 µm.

D. EVI/WLS-V5 accumulates in the ER after transfection of siUBE2N. HeLa cells were transfected with siUBE2N and seeded on cover glasses 24 h later. The next day, cells were additionally transfected with EVI/WLS-V5. Again 24 h later, cells were fixed with 4%

paraformaldehyde/PBS and stained for V5 (EVI/WLS) and SERCA2 as ER marker. Images were acquired using a Zeiss motorised inverted Observer.Z1 microscope with the ZEISS ZEN (blue edition) software. A total of n = 84 (siLuciferase) and n = 88 (siUBE2N) cells were imaged in four independent experiments and their corrected total cell fluorescence (CTCF) was analysed using Fiji (Fiji is just ImageJ). **D'** Shown are representative images where brightness and contrast were adjusted. Scale bar is 10 µm. **D''** Each dot represents an individual cell and replicates are indicated by dot colour. Two-Sample Wilcoxon test, two-sided, * p-value = 3.479e-08

Journal of Cell Science • Supplementary information

Table S1. Summarised results of the low-throughput screen

Observed phenotypes on EVI/WLS protein abundance in HEK293T and A375 cells after knock-down of indicated target genes. Data derived from this study or from Glaeser et al., 2018. - no effect; + weak or variable effects; ++ strong upregulation

	Gene	Abbreviation	NCBI gene ID	UniProtKB	Cell line	Results	Reference
**Substrate recognition**	ERLEC1	Endoplasmic reticulum lectin 1	27248	Q96DZ1	HEK293T	-/+	this work
	ERLIN1/SPFH1	ER Lipid Raft Associated 1	10613	O75477	HEK293T	-	this work
	ERLIN2/SPFH2	ER Lipid Raft Associated 2	11160	O94905	HEK293T	+/++	this work
					A375	+	this work
	OS9/ERLEC2	OS9 Endoplasmic Reticulum Lectin	10956	Q13438	HEK293T	-	this work
	SEL1L/HRD3	SEL1L Adaptor Subunit Of ERAD E3 Ubiquitin Ligase	6400	Q9UBV2	HEK293T	+	this work
					A375	+	this work
**E2 ubiquitin conjugating enzymes**	UBE2D1	Ubiquitin Conjugating Enzyme E2 D1	7321	P51668	HEK293T	-	this work
	UBE2G1	Ubiquitin Conjugating Enzyme E2 G1	7326	P62253	HEK293T	-	this work
	UBE2G2	Ubiquitin Conjugating Enzyme E2 G2	7327	P60604	HEK293T	-	Glaeser et al., 2018
					A375	-/+	this work
	UBE2J1	Ubiquitin Conjugating Enzyme E2 J1	51465	Q9Y385	HEK293T	-	Glaeser et al., 2018
					A375	-	this work
	UBE2J2	Ubiquitin Conjugating Enzyme E2 J2	118424	Q8N2K1	HEK293T	++	Glaeser et al., 2018
					A375	++	this work
	UBE2K	Ubiquitin Conjugating Enzyme E2 K	3093	P61086	HEK293T	++	this work
					A375	+	this work
	UBE2N/UBC13	Ubiquitin Conjugating Enzyme E2 N	7334	P61088	HEK293T	+/++	this work
					A375	+	this work
	UBE2U	Ubiquitin Conjugating Enzyme E2 U	148581	Q5VVX9	HEK293T	-	Glaeser et al., 2018
					A375	-	this work
**E3 ubiquitin ligases**	AMFR/GP78/RNF45	Autocrine Motility Factor Receptor	267	Q9UKV5	HEK293T	-/+	Glaeser et al., 2018
					A375	+	this work
	CGRRF1/RNF197	Cell Growth Regulator With Ring Finger Domain 1	10668	Q99675	HEK293T	++	Glaeser et al., 2018
					A375	++	this work
	HRD1/SYVN1	Synoviolin 1	84447	Q86TM6	HEK293T	-	Glaeser et al., 2018
					A375	-	this work
	MARCH4/RNF174	Membrane Associated Ring-CH-Type Finger 4	57574	Q9P2E8	HEK293T	-	Glaeser et al., 2018
					A375	+/++	this work
	MARCH6/TEB4/ RNF176	Membrane Associated Ring-CH-Type Finger 6	10299	O60337	HEK293T	+	Glaeser et al., 2018
					A375	-/+	this work
	RNF5	Ring Finger Protein 5	6048	Q99942	HEK293T	-/+	Glaeser et al., 2018
					A375	+	this work
	RNF24	Ring Finger Protein 24	11237	Q9Y225	HEK293T	-/+	this work
	RNF122	Ring Finger Protein 122	79845	Q9H9V4	HEK293T	-/+	this work
	RNF128	Ring Finger Protein 128	79589	Q8TEB7	HEK293T	-	Glaeser et al., 2018
					A375	-/+	this work
	RNF139/TRC8	Ring Finger Protein 139	11236	Q8WU17	HEK293T	+	Glaeser et al., 2018
					A375	-	this work
	RNF170	Ring Finger Protein 170	81790	Q96K19	HEK293T	-	Glaeser et al., 2018
					A375	-	this work
	STUB1/CHIP	STIP1 homology and U-box containing protein 1	10273	Q9UNE7	HEK293T	-	this work
					A375	-/+	this work
	UBE4B/UFD2	Ubiquitylation Factor E4B	10277	O95155	HEK293T	-	this work

Journal of Cell Science • Supplementary information

	Gene	Abbreviation	NCBI gene ID	UniProtKB	Cell line	Results	Reference
**Retrotrans-location/Dis-location**	DERL1	Derlin 1	79139	Q9BUN8	HEK293T	+	this work
					A375	-	this work
	DERL2	Derlin 2	51009	Q9GZP9	HEK293T	+	this work
	DERL3	Derlin 3	91319	Q96Q80	HEK293T	+/++	this work
	FAF2/ETEA/UBXD8	Fas Associated Factor Family Member 2	23197	Q96CS3	HEK293T	+/++	this work
					A375	+/++	this work
	HM13/SPP	Histocompatibility Minor 13/ Signal Peptide Peptidase	81502	Q8TCT9	HEK293T	+	this work
					A375	-	this work
	NPLOC4/NPL4	NPL4 Homolog, Ubiquitin Recognition Factor	55666	Q8TAT6	HEK293T	+/++	this work
	RHBDD1/RHBDL4	Rhomboid Domain Containing 1	84236	Q8TEB9	HEK293T	+	this work
					A375	-	this work
	UBAC2	UBA Domain Containing 2	337867	Q8NBM4	HEK293T	+	this work
	UBXN4/ERASIN/ UBXD2	UBX Domain Protein 4	23190	Q92575	HEK293T	+/++	this work
					A375	+	this work
	UBXN6/UBXD1	UBX Domain Protein 6	80700	Q9BZV1	HEK293T	+/++	this work
	UFD1/UFD1L	Ubiquitin Recognition Factor In ER Associated Degradation 1	7353	Q92890	HEK293T	+/++	this work
	VCP/P97/CDC48	Valosin Containing Protein	7415	P55072	HEK293T	++	Glaeser et al., 2018
					A375	++	this work
**Delivery to the proteasome**	BAG6/BAT3/SCYTHE	BAG Cochaperone 6	7917	P46379	HEK293T	-	this work
	HERPUD1/HERP	Homocysteine Inducible ER Protein With Ubiquitin Like Domain 1	9709	Q15011	HEK293T	+	this work
	NGLY/PNG1	N-Glycanase 1	55768	Q96IV0	HEK293T	+	this work
	RAD23A	RAD23 Homolog A, Nucleotide Excision Repair Protein	5886	P54725	HEK293T	-/+	this work
	RAD23B	RAD23 Homolog B, Nucleotide Excision Repair Protein	5887	P54727	HEK293T	-/+	this work
	TMUB1/HOPS	Transmembrane And Ubiquitin Like Domain Containing 1	83590	Q9BVT8	HEK293T	-	this work
	TMUB2	Transmembrane And Ubiquitin Like Domain Containing 2	79089	Q71RG4	HEK293T	+/++	this work
	UBQLN2/DSK2	Ubiquilin 2	29978	Q9UHD9	HEK293T	-	this work
**Deubiquityl-ating enzymes**	ATXN3	Ataxin 3	4287	P54252	HEK293T	-	this work
	USP13	Ubiquitin Specific Peptidase 13	8975	Q92995	HEK293T	-	this work
	USP19	Ubiquitin Specific Peptidase 19	10869	O94966	HEK293T	+	this work
	USP25	Ubiquitin Specific Peptidase 25	29761	Q9UHP3	HEK293T	-	this work
	USP50	Ubiquitin Specific Peptidase 50	373509	Q70EL3	HEK293T	+/++	this work
	VCPIP1	Valosin Containing Protein Interacting Protein 1	80124	Q96JH7	HEK293T	-	this work
	YOD1/OTUD2	YOD1 Deubiquitylase	55432	Q5VVQ6	HEK293T	-	this work

Journal of Cell Science • Supplementary information

Table S2. siRNA references used in this study

Target gene	siRNA number	Manufacturer	Reference
EVI/WLS	1	Ambion	s36745
EVI/WLS	3	Ambion	s36747
siGENOME Non-Targeting siRNA Pool #1	/	Dharmacon	D-001206-13-20
siLuciferase/ RLuc Duplex siRNA	/	Dharmacon	P-002070-01-20
AMFR/GP78	1	Dharmacon	D-006522-01
AMFR/GP78	2	Dharmacon	D-006522-02
AMFR/GP78	3	Dharmacon	D-006522-03
AMFR/GP78	4	Dharmacon	D-006522-04
ATXN3	1	Dharmacon	D-012013-01
ATXN3	2	Dharmacon	D-012013-02
ATXN3	3	Dharmacon	D-012013-03
ATXN3	4	Dharmacon	D-012013-04
BAG6	1	Dharmacon	D-005062-01
BAG6	2	Dharmacon	D-005062-02
BAG6	3	Dharmacon	D-005062-03
BAG6	4	Dharmacon	D-005062-04
CGRRF1	1	Dharmacon	D-006933-01
CGRRF1	2	Dharmacon	D-006933-02
CGRRF1	3	Dharmacon	D-006933-03
CGRRF1	4	Dharmacon	D-006933-04
DERL1	2	Dharmacon	D-010733-02
DERL1	3	Dharmacon	D-010733-03
DERL1	4	Dharmacon	D-010733-04
DERL1	18	Dharmacon	D-010733-18
DERL2	1	Dharmacon	D-010576-01
DERL2	2	Dharmacon	D-010576-02
DERL2	3	Dharmacon	D-010576-03
DERL2	4	Dharmacon	D-010576-04
DERL3	4	Dharmacon	D-032237-04
DERL3	5	Dharmacon	D-032237-05
DERL3	6	Dharmacon	D-032237-06
DERL3	7	Dharmacon	D-032237-07
ERLIN1	2	Dharmacon	D-015639-02
ERLIN1	18	Dharmacon	D-015639-18
ERLIN1	19	Dharmacon	D-015639-19
ERLIN1	20	Dharmacon	D-015639-20
ERLIN2/SPFH2	2	Dharmacon	D-017943-02
ERLIN2/SPFH2	3	Dharmacon	D-017943-03
ERLIN2/SPFH2	4	Dharmacon	D-017943-04
ERLIN2/SPFH2	5	Dharmacon	D-017943-05
FAF2/ETEA/UBXD8	1	Dharmacon	D-010649-01
FAF2/ETEA/UBXD8	3	Dharmacon	D-010649-03
FAF2/ETEA/UBXD8	4	Dharmacon	D-010649-04
FAF2/ETEA/UBXD8	17	Dharmacon	D-010649-17
HERPUD1/HERP	1	Dharmacon	D-020918-01
HERPUD1/HERP	2	Dharmacon	D-020918-02
HERPUD1/HERP	3	Dharmacon	D-020918-03
HERPUD1/HERP	4	Dharmacon	D-020918-04
HM13/SPP	4	Dharmacon	D-005896-4
HM13/SPP	5	Dharmacon	D-005896-5
HM13/SPP	6	Dharmacon	D-005896-6
HM13/SPP	19	Dharmacon	D-005896-19
MARCH4	2	Dharmacon	D-023172-02
MARCH4	3	Dharmacon	D-023172-03
MARCH4	5	Dharmacon	D-023172-05
MARCH4	6	Dharmacon	D-023172-06
MARCH6	1	Dharmacon	D-006925-01
MARCH6	2	Dharmacon	D-006925-02
MARCH6	3	Dharmacon	D-006925-03
MARCH6	4	Dharmacon	D-006925-04
NGLY/PNG1	1	Dharmacon	D-016457-01
NGLY/PNG1	2	Dharmacon	D-016457-02
NGLY/PNG1	3	Dharmacon	D-016457-03
NGLY/PNG1	4	Dharmacon	D-016457-04
NPLOC4/NPL4	2	Dharmacon	D-020796-02
NPLOC4/NPL4	3	Dharmacon	D-020796-03
NPLOC4/NPL4	4	Dharmacon	D-020796-04
NPLOC4/NPL4	17	Dharmacon	D-020796-17

Journal of Cell Science • Supplementary information

Target gene	siRNA number	Manufacturer	Reference
PORCN	1	Dharmacon	D-009613-01
PORCN	2	Dharmacon	D-009613-02
PORCN	3	Dharmacon	D-009613-03
PORCN	4	Dharmacon	D-009613-04
RAD23B	1	Dharmacon	D-011759-01
RAD23B	2	Dharmacon	D-011759-02
RAD23B	3	Dharmacon	D-011759-03
RAD23B	4	Dharmacon	D-011759-04
RHBDD1/RHBDL4	1	Dharmacon	D-019378-01
RHBDD1/RHBDL4	2	Dharmacon	D-019378-02
RHBDD1/RHBDL4	3	Dharmacon	D-019378-03
RHBDD1/RHBDL4	4	Dharmacon	D-019378-04
RNF128	1	Dharmacon	D-007061-01
RNF128	4	Dharmacon	D-007061-04
RNF128	17	Dharmacon	D-007061-17
RNF128	18	Dharmacon	D-007061-18
RNF139/TRC8	1	Dharmacon	D-006942-01
RNF139/TRC8	2	Dharmacon	D-006942-02
RNF139/TRC8	4	Dharmacon	D-006942-04
RNF139/TRC8	17	Dharmacon	D-006942-17
RNF170	1	Dharmacon	D-007078-01
RNF170	2	Dharmacon	D-007078-02
RNF170	3	Dharmacon	D-007078-03
RNF170	4	Dharmacon	D-007078-04
RNF5	1	Dharmacon	D-006558-01
RNF5	2	Dharmacon	D-006558-02
RNF5	3	Dharmacon	D-006558-03
RNF5	18	Dharmacon	D-006558-18
SEL1L	2	Dharmacon	D-004885-02
SEL1L	3	Dharmacon	D-004885-03
SEL1L	4	Dharmacon	D-004885-04
SEL1L	5	Dharmacon	D-004885-05
STUB1/CHIP	2	Dharmacon	D-007201-02
STUB1/CHIP	3	Dharmacon	D-007201-03
STUB1/CHIP	5	Dharmacon	D-007201-05
STUB1/CHIP	6	Dharmacon	D-007201-06
SYFN/HRD1	1	Dharmacon	D-007090-01
SYFN/HRD1	2	Dharmacon	D-007090-02
SYFN/HRD1	3	Dharmacon	D-007090-03
SYFN/HRD1	4	Dharmacon	D-007090-04
TMUB2	1	Dharmacon	D-014307-01
TMUB2	3	Dharmacon	D-014307-03
TMUB2	4	Dharmacon	D-014307-04
TMUB2	18	Dharmacon	D-014307-18
UBAC2	1	Dharmacon	D-107914-01
UBAC2	2	Dharmacon	D-107914-02
UBAC2	3	Dharmacon	D-107914-03
UBAC2	4	Dharmacon	D-107914-04
UBE2G1	1	Dharmacon	D-010154-01
UBE2G1	2	Dharmacon	D-010154-02
UBE2G1	4	Dharmacon	D-010154-04
UBE2G1	5	Dharmacon	D-010154-05
UBE2G2	1	Dharmacon	D-009095-01
UBE2G2	2	Dharmacon	D-009095-02
UBE2G2	3	Dharmacon	D-009095-03
UBE2G2	5	Dharmacon	D-009095-05
UBE2J1	1	Dharmacon	D-007266-01
UBE2J1	3	Dharmacon	D-007266-03
UBE2J1	19	Dharmacon	D-007266-19
UBE2J1	20	Dharmacon	D-007266-20
UBE2J2	1	Dharmacon	D-008614-01
UBE2J2	2	Dharmacon	D-008614-02
UBE2J2	4	Dharmacon	D-008614-04
UBE2J2	18	Dharmacon	D-008614-18
UBE2K	17	Dharmacon	D-009431-17
UBE2K	18	Dharmacon	D-009431-18
UBE2K	19	Dharmacon	D-009431-19
UBE2K	20	Dharmacon	D-009431-20
UBE2N	1	Dharmacon	D-003920-01
UBE2N	2	Dharmacon	D-003920-02
UBE2N	4	Dharmacon	D-003920-04
UBE2N	5	Dharmacon	D-003920-05

Journal of Cell Science • Supplementary information

Target gene	siRNA number	Manufacturer	Reference
UBE2U	1	Dharmacon	D-008998-01
UBE2U	2	Dharmacon	D-008998-02
UBE2U	3	Dharmacon	D-008998-03
UBE2U	4	Dharmacon	D-008998-04
UBE2V1	2	Dharmacon	D-010064-02
UBE2V1	21	Dharmacon	D-010064-21
UBE2V1	22	Dharmacon	D-010064-22
UBE2V1	23	Dharmacon	D-010064-23
UBE2V2	1	Dharmacon	D-008823-01
UBE2V2	2	Dharmacon	D-008823-02
UBE2V2	3	Dharmacon	D-008823-03
UBE2V2	4	Dharmacon	D-008823-04
UBE4A	1	Dharmacon	D-007200-01
UBE4A	2	Dharmacon	D-007200-02
UBE4A	3	Dharmacon	D-007200-03
UBE4A	4	Dharmacon	D-007200-04
UBXN4/ERASIN/UBXD2	3	Dharmacon	D-014184-03
UBXN4/ERASIN/UBXD2	4	Dharmacon	D-014184-04
UBXN4/ERASIN/UBXD2	17	Dharmacon	D-014184-17
UBXN4/ERASIN/UBXD2	18	Dharmacon	D-014184-18
UBXN6/UBXD1	1	Dharmacon	D-008785-01
UBXN6/UBXD1	2	Dharmacon	D-008785-02
UBXN6/UBXD1	3	Dharmacon	D-008785-03
UBXN6/UBXD1	4	Dharmacon	D-008785-04
UFD1L/UFD1	2	Dharmacon	D-017918-02
UFD1L/UFD1	3	Dharmacon	D-017918-03
UFD1L/UFD1	4	Dharmacon	D-017918-04
UFD1L/UFD1	17	Dharmacon	D-017918-17
USP13	1	Dharmacon	D-006064-01
USP13	2	Dharmacon	D-006064-02
USP13	3	Dharmacon	D-006064-03
USP13	4	Dharmacon	D-006064-04
USP19	3	Dharmacon	D-006068-03
USP19	4	Dharmacon	D-006068-04
USP19	5	Dharmacon	D-006068-05
USP19	6	Dharmacon	D-006068-06
USP25	2	Dharmacon	D-006074-02
USP25	3	Dharmacon	D-006074-03
USP25	4	Dharmacon	D-006074-04
USP25	5	Dharmacon	D-006074-05
USP50	2	Dharmacon	D-031837-2
USP50	3	Dharmacon	D-031837-3
USP50	4	Dharmacon	D-031837-4
USP50	17	Dharmacon	D-031837-17
VCP	5	Dharmacon	D-008727-05
VCP	6	Dharmacon	D-008727-06
VCP	7	Dharmacon	D-008727-07
VCP	8	Dharmacon	D-008727-08
VCPIP1	1	Dharmacon	D-019137-01
VCPIP1	2	Dharmacon	D-019137-02
VCPIP1	3	Dharmacon	D-019137-03
VCPIP1	4	Dharmacon	D-019137-04
YOD1	1	Dharmacon	D-027369-01
YOD1	2	Dharmacon	D-027369-02
YOD1	3	Dharmacon	D-027369-03
YOD1	4	Dharmacon	D-027369-04
**siRNAs from Dharmacon Genomewide 96 well plates MTP**			
ERLEC1	/	Dharmacon	M-010658-00
OS9	/	Dharmacon	M-010811-00
RAD23A	/	Dharmacon	M-005231-00
RNF122	/	Dharmacon	M-007068-00
RNF24	/	Dharmacon	M-006943-00
TMUB1	/	Dharmacon	M-018578-00
TMUB2	/	Dharmacon	M-014307-00
UBAC2	/	Dharmacon	M-017914-00
UBE2D1	/	Dharmacon	M-009387-01
UBQLN2	/	Dharmacon	M-013566-00
UFD2/UBE4B	/	Dharmacon	M-007202-01

Journal of Cell Science • Supplementary information

Table S3. Sequences of primers used for RT-qPCR

Target mRNA	Forward primer sequence (5' -> 3')	Reverse primer sequence (5' -> 3')	Probe	Probe Reference
ACTB	CCAACCGCGAGAAGATGA	CCAGAGGCGTACAGGGATAG	#64	4688635001
AXIN2	GCTGACGGATGATTCCATGT	ACTGCCCACACGATAAGGAG	#56	4688538001
ERLIN2/SPFH2	GGAAGAAGGCGCTCATTG	TGAAATCTTCTCAGTCTCCTTC	#29	4687612001
EVI/WLS	TCATGGTATTTCAGGTGTTTCG	GCATGAGGAACTTGAACCTAAAA	#38	4687965001
FAF2/ETEA/UBXD8	GAAGGAGGAGGAGGTGCAA	TCCTTTCCTTTTCCTCCTGTAA	#82	4689054001
GAPDH	AGCCACATCGCTCAGACAC	GCCCAATACGACCAAATCC	#60	4688589001
NPLOC4	CGGTTTACATCAATAGAAACAAGACT	AACAACAAATCGCCATGCTT	#25	4686993001
PORCN	GCTACTGCAAGGCTGTCTCC	GCTTCAGGTAGGATGGCAAC	#3	4685008001
SDHA	GGACCTGGTTGGTCTTTGGTC	CCAGCGTTTGGTTTAATTGG	#80	4689038001
UBE2K	AGGACCTCCAGACACACCAT	CGGACCTTAGGGGGATTAAA	#69	4688686001
UBE2N	CGCAGGATCATCAAGGAAA	AAATAACGGGCGTTGCTCT	#72	4688953001
UBE2V1	GGGCCTCCAAGATTTCAGTT	AAGGCTGTATATTCGGTTTTCATAA	#82	4689054001
UBE2V2	ACAAGGTGGACAGGCATGAT	TCTGGGTATTTAGGTCCACATTC	#49	4688104001
UBXN4/ERASIN/UBXD2	CGCTTCGGTGGTACTGTTG	TCCCAACAAGGTCCAAATGT	#4	4685016001
UBXN6	CCTGGACAACATCCACCTG	AGGCAGTTAATGCGCTCCT	#63	4688627001
UFD1l/UFD1	CAGCATGAGGAGTCGACAGA	CCAGTCTATTGCCAGATCCAG	#67	4688660001
VCP	AGAGGCAGACAAACCCATCA	AGTGATCTCGACGGATCTCAG	#35	4687680001
WNT5A	CAGTTCAAGACCGTGCAGAC	ACGATCTCCGTGCACTTCTT	#59	4688562001

Journal of Cell Science • Supplementary information

Table S4. Antibodies, TUBEs, and reagents for immunoprecipitations used in this study

End. – endogenous; IF – Immunofluorescence staining; IP - Immunoprecipitation; WB – Western blot

Primary antibodies

Target protein	Reference	LOT	Clonality	Dilution (Application)	Host	Supplier
β-ACTIN	ab6276	GR3185620-6	monoclonal (AC-15)	1/ 10 000 (WB)	mouse	Abcam
β-ACTIN	#4967	8	polyclonal	1/ 1 000 (WB)	rabbit	Cell Signaling Technology
ERLIN2/SPFH2	EB06896	G2 N201107	polyclonal	1/ 1 000 (WB)	goat	Everest Biotech
ERLIN2/SPFH2	#2959	2	polyclonal	1/ 50 (end. IP)	rabbit	Cell Signaling Technology
EVI/WLS	655902	B225314	monoclonal (YJ5)	1/250 - 1/ 1 000 (WB)	mouse	BioLegend
FAF2/ETEA/UBXD8	GTX14759	821702089	polyclonal	1/ 1 000 (WB), 1/ 250 (IF)	goat	GeneTex
FAF2/ETEA/UBXD8	#34945	1	monoclonal (D8H6D)	1/ 200 (end. IP)	rabbit	Cell Signaling Technology
FLAG	F1804	SLBG5673V	monoclonal (M2)	1/ 1 000 (WB)	mouse	Sigma-Aldrich
FLAG	F7425	078M4886V	polyclonal	1/ 8 000 (WB)	rabbit	Sigma-Aldrich
HSC70	sc-7298	D0218	monoclonal (clone B-6)	1/ 2 000 (WB)	mouse	Santa Cruz Biotechnology
K48-linkage Specific Polyubiquitin	#8081	2	monoclonal (D9D5)	1/ 100 000 (WB)	rabbit	Cell Signaling Technology
K63-linkage Specific Polyubiquitin	#5621	5	monoclonal (D7A11)	1/ 1 000 (WB)	rabbit	Cell Signaling Technology
SEL1L	ab78298	GR215038-1	polyclonal	1/1 000 (WB)	rabbit	Abcam
SERCA2 ATPase	ab2861	GR283954-6	monoclonal (2A7-A1)	1/ 500 (IF)	mouse	Abcam
α-TUBULIN	#2144	6	polyclonal	1/ 3 000 (WB)	rabbit	Cell Signaling Technology
UBE2K/E2-25K	MAB6609	CFHC0118071	monoclonal (701316)	1/ 1 000 (WB)	mouse	R&D Systems
UBE2N	#6999	1	monoclonal (D2A1)	1/ 1 000 (WB)	rabbit	Cell Signaling Technology
V5 Epitope Tag	600-401-378	29087	polyclonal	1/ 500 (IF)	rabbit	Rockland
VCP/P97	ab11433	GR264288-5	monoclonal (5)	1/ 100 000 (WB)	mouse	Abcam
VINCULIN	AB6039	*unknown*	polyclonal	1/ 150 000 (WB)	rabbit	Merck Millipore
WNT3	GTX128100	41171	polyclonal	1/ 1 000 (WB)	rabbit	GeneTex
WNT5A/B	#2530	4	monoclonal (C27E8)	1/ 1 000 (WB)	rabbit	Cell Signaling Technology

Horse-radish peroxidase (HRP)-coupled antibodies for Western blotting

Specificity	Reference	Host	Clonality	Conjugate	Dilution	Supplier
β-ACTIN	SC47778 HRP	mouse	monoclonal (C4)	HRP	1/ 5 000	Santa Cruz Biotechnology
Anti-Goat IgG	6160-05	rabbit	polyclonal	HRP	1/ 5 000	SouthernBiotech
Anti-Mouse IgG (H+L)	AB_10015289	goat	polyclonal	HRP	1/ 10 000	Jackson ImmunoResearch
Anti-Rabbit IgG (H+L)	AB_2313567	goat	polyclonal	HRP	1/ 10 000	Jackson ImmunoResearch

Fluorescence-protein coupled antibodies for immunofluorescence staining

Name	Conjugate	Dilution	Reference	Supplier
Donkey anti-Rabbit IgG (H+L)	Alexa Fluor 488	1/ 500	A-21206	Thermo Fisher Scientific
Donkey anti-Rabbit IgG (H+L)	Alexa Fluor 555	1/ 500	A-31572	Thermo Fisher Scientific
Donkey anti-Mouse IgG (H+L)	Alexa Fluor 647	1/ 500	A-31571	Thermo Fisher Scientific
Donkey anti-Goat IgG (H+L)	Alexa Fluor 647	1/ 500	A-21447	Thermo Fisher Scientific

Tandem Ubiquitin Binding Entities (TUBEs), immunoprecipitation, and control reagents

Name	Specificity	Conjugate	Reference	Supplier
TUBE Control	Control	Agarose beads	UM400	LifeSensors
TUBE1	Pan-ubiquitin	Magnetic beads	UM401M	LifeSensors
TUBE2	Pan-ubiquitin	Agarose beads	UM402	LifeSensors
K48-TUBE	K48-linked ubiquitin	FLAG-tag	UM607	LifeSensors
K63-TUBE	K63-linked ubiquitin	FLAG-tag	UM604	LifeSensors
M2 AFFINITY GEL	Anti-FLAG	Agarose beads	A2220	Sigma-Aldrich
Monoclonal Anti-HA-Agarose	Anti-HA	Agarose beads	A2095	Sigma-Aldrich
Dynabeads Protein G	Immunoglobulin Fc-region	Magnetic beads	10004D	Life Technologies
BLUE SEPHAROSE 6 Fast Flow	General protein purification	Sepharose	17-0948-01	GE Healthcare
Normal Rabbit Immunoglobulin	Control	N/A	500-P00	PeproTech

Journal of Cell Science • Supplementary information
