# Supplementary material for: EVI/WLS function is regulated by ubiquitylation and is linked to ER-associated degradation by ERLIN2
Source: J Cell Sci. 2021 Aug 18;134(16):jcs257790. doi: 10.1242/jcs.257790 (PMC8435288; doi:10.1242/jcs.257790)
Supplement: Reviewer comments [file joces-134-257790_review_history.pdf]

EVI/WLS function is regulated by ubiquitylation and linked to ER-associated degradation by ERLIN2

Lucie M. Wolf, Annika M. Lambert, Julie Haenlin and Michael Boutros
DOI: 10.1242/jcs.257790

Editor: John Heath

Review timeline
Original submission: 30 November 2020
Editorial decision: 4 January 2021
First revision received: 8 June 2021
Accepted: 12 July 2021

Original submission

First decision letter

MS ID#: JOCES/2020/257790

MS TITLE: EVI/WLS function is regulated by ubiquitination and linked to ER-associated degradation by ERLIN2

AUTHORS: Michael Boutros, Lucie Wolf, Annika Lambert, and Julie Haenlin

ARTICLE TYPE: Research Article

We have now reached a decision on the above manuscript.

To see the reviewers' reports and a copy of this decision letter, please go to: https://submit-jcs.biologists.org and click on the 'Manuscripts with Decisions' queue in the Author Area. (Corresponding author only has access to reviews.)

As you will see, the reviewers raise a number of substantial criticisms that prevent me from accepting the paper at this stage. They suggest, however, that a revised version might prove acceptable, if you can address their concerns. If you think that you can deal satisfactorily with the criticisms on revision, I would be pleased to see a revised manuscript. We would then return it to the reviewers.

We are aware that you may be experiencing disruption to the normal running of your lab that makes experimental revisions challenging. If it would be helpful, we encourage you to contact us to discuss your revision in greater detail. Please send us a point-by-point response indicating where you are able to address concerns raised (either experimentally or by changes to the text) and where you will not be able to do so within the normal timeframe of a revision. We will then provide further guidance. Please also note that we are happy to extend revision timeframes as necessary.

Please ensure that you clearly highlight all changes made in the revised manuscript. Please avoid using 'Tracked changes' in Word files as these are lost in PDF conversion. Instead please use yellow shading or different colour font to denote changes in your revised manuscript.

I should be grateful if you would also provide a point-by-point response detailing how you have dealt with the points raised by the reviewers in the 'Response to Reviewers' box. Please attend to

© 2021. Published by The Company of Biologists under the terms of the Creative Commons Attribution License (https://creativecommons.org/licenses/by/4.0/).

all of the reviewers' comments. If you do not agree with any of their criticisms or suggestions please explain clearly why this is so.

Reviewer 1

Advance summary and potential significance to field

L. Wolf et al. demonstrated that the Wnt secretory factor WLS/ EVI/ GPR177 is differentially ubiquitinated through the E2 conjugating enzymes UBE2J2, UBE2N and UBE2K. They were not able to identify the E3 ligases specific for WLS, but showed that HRD1/ SYVN are not necessary for WLS ubiquitination. They perform their experiments on HEK293T as well as the melanoma cells A357, potentially demonstrating novel regulatory mechanisms of Wnt secretory pathway.

Comments for the author

Major points:
The data presented is extremely interesting but I would suggest a few modifications/ experiments that could improve the quality of the work.

The immunoprecipitation data is performed on cells overexpressing the different plasmids. It would be interesting to reproduce some of the key findings on endogenous WLS, as the antibodies seem to work properly.

As WLS expression is known to regulate melanoma cells proliferation (https://doi.org/10.1002/emmm.201201486), please check how knockdown of VCP and UBE2N affect the proliferation (or colony formation) of the melanoma cells.

According to ubibrowser (http://ubibrowser.ncpsb.org/ubibrowser/strict/networkview/networkview/name/Q5T9L3/jobId/ubibrowse-I2020-12-14-98165-1607940581), it is predicted that WLS interacts with different E3 ligases. Please validate whether the knockdown of the E3 ligases expressed in the melanoma cells (such as ITCH) are required for WLS ubiquitination

Minor ponts:
Please add the statistical analysis to the different qPCR data analyses: Fig 2c, f, i, l etc.
I would replace "mini screen" by "low throughput screen" in Supplementary Figure 1.

Reviewer 2

Advance summary and potential significance to field

In this study, Wolf et al. identify a number of novel components that are involved in the turnover of the EVI/WLS protein that performs a central role in WNT secretion. Using a well-designed siRNA-based screen, various ERAD-linked proteins are identified that regulate cellular EVI/WLS protein levels.
Furthermore, a link is reported between EVI/WLS levels and WNT expression and secretion, indicating that interaction of these proteins mediates their reciprocate stabilisation. Follow-up experiments indicate that the newly identified components regulate EVI/WLS levels via ubiquitination-dependent mechanisms. These results thus shed light on how components of major cellular ubiquitin-mediated degradation pathways jointly operate to control WNT signalling, a key developmental pathway.

Comments for the author

In general, data are presented in a clear and comprehensive manner. The screen set-up appears robust and well-controlled, indicating regulation of EVI/WLS at the posttranslational level. The follow-up work, aimed at the validation of novel candidates and insight in the underlying

© 2021. Published by The Company of Biologists under the terms of the Creative Commons Attribution License (https://creativecommons.org/licenses/by/4.0/).

mechanisms, however, appears a weaker part of this study. Moreover, data are overinterpreted at a few places.

Key points of criticism include a lack of proof for direct ubiquitination of EVI/WLS, and the absence of linking key protein-protein interactions and EVI/WLS degradation steps to subcellular locations. Finally, the consequences of chemical inhibitors with broad cellular impact should be interpreted more carefully.

Specific points:
Following the first screen in HEK293T cells, candidate genes are evaluated for regulating EVI/WLS levels in A375 cells. However, only previously reported candidates (published) are confirmed here, while newly identified genes are not included. Why not? Demonstration of these observations in 2 cell lines would strengthen the initial findings. (siRNAs are applied in A375 cells in Figure 6 but effects on EVI/WLS levels compared to siLuc are not clear in all cases.

Plus, some of these experiments include the use of MG132, which is not an appropriate comparison)
The manuscript states at multiple places the detection of 'ubiquitinated EVI/WLS', or 'EVI/WLS modified with ubiquitin'. However, evidence for direct modification of the protein is not provided. While the detection of higher MW species for EVI/WLS indeed is a nice lead, it is by no means a direct proof for ubiquitin attachment. Furthermore, as pulldown experiments (using TUBE or HA-tagged Ub) are seemingly not performed under denaturing conditions, it cannot be excluded that EVI/WLS co-aggregates with other Ub-modified proteins to co-migrate at higher MW regions on the gel.

Here, mass spec-based methods would help to demonstrate direct modification of EVI/WLS with ubiquitin. In fact, on page 18 it is mentioned that 'Publicly available MS data report ubiquitination at several lysine residues' (link should be provided), indicating that methods are available. In addition, can the authors express a lysine-less EVI/WLS protein in EVI/WLS-ko cells and demonstrate that this variant is insensitive to the identified siRNAs?
Page 13: "…the accumulation of K63-ubiquitination of EVI/WLS after MG132 treatment indicates proteasomal degradation." Treatment with proteasomal inhibitors will induce a plethora of changes on overall protein levels and activity, which may cause indirect effects on protein modification and stability, in particular when consequences are evaluated at late timepoints (24h) as indicated in the methods section. Thus, these findings should be interpreted with care. As K63-ubiquitination generally targets for endo-lysosomal turnover of proteins, treatment with bafilomycin, a lysosomal inhibitor, should be included for comparison. In addition, control experiments using a lysine-dead EVI/WLS variant would help to draw more firm conclusions.

As the observed effects on EVI/WLS levels likely link to different cellular degradation pathways (qualitative/quantitative ERAD, endo-lysosomal degradation), this point should be better clarified and discussed. A correlation between key protein-protein interactions and degradation steps with subcellular localization would be required to strengthen this poiny. Where do the identified components colocalize with EVI/WLS, and where does the EVI/WLS protein accumulate in various knock-down conditions?

Minor points:
In the results section, a 'focused siRNA and Western blot screen' is mentioned (page 5, top). Here, it would be helpful to provide more information to the reader in the text; which types of genes were screened and what was the rationale?

Figure 1: The DUB enzyme USP50 as well as the proteasome delivery component TMUB2 are also picked up as relevant targets but these are not discussed in the text. What do these findings mean and why were these not discussed or followed up?
Reference is made to the phenotype of the UBC13 knockout in c.elegans, where EVI/WLS is diverted to lysosomes (Zhang et al 2018). How does this match with observed increase in EVI/WLS protein levels?

Typos:
Abstract: '….regulatory ER-associated degradation (ERAD) has been implicated (in) the production of Wnt Proteins.'

© 2021. Published by The Company of Biologists under the terms of the Creative Commons Attribution License (https://creativecommons.org/licenses/by/4.0/).

Last sentence of the introduction: '….ERAD, and further emphasises the link between ubiquitination and WNT signalling'

First revision

Author response to reviewers' comments

We would like to thank both reviewers for their constructive comments and suggestions and have revised the manuscript accordingly. We have outlined below how we addressed the reviewer's comments and discuss which we could not address given the current state of the art or circumstances.

Reviewer 1

"Advance Summary and Potential Significance to Field:
L. Wolf et al. demonstrated that the Wnt secretory factor WLS/ EVI/ GPR177 is differentially ubiquitinated through the E2 conjugating enzymes UBE2J2, UBE2N and UBE2K. They were not able to identify the E3 ligases specific for WLS, but showed that HRD1/ SYVN are not necessary for WLS ubiquitination. They perform their experiments on HEK293T as well as the melanoma cells A357, potentially demonstrating novel regulatory mechanisms of Wnt secretory pathway."

Reviewer 1 Comments for the Author:

"Major points:

The data presented is extremely interesting but I would suggest a few modifications/ experiments that could improve the quality of the work."

We thank the reviewer for the positive comment.

"The immunoprecipitation data is performed on cells overexpressing the different plasmids. It would be interesting to reproduce some of the key findings on endogenous WLS, as the antibodies seem to work properly."

We fully agree that immunoprecipitation data on endogenous proteins is most interesting and would like to point out that the detected EVI/WLS in the respective experiments (Figure 3, Figure S4) was endogenous and not overexpressed.
However, as its interaction partners were overexpressed (FLAG-tagged constructs of ERLIN2, FAF2, UBXN4), we have now repeated the immunoprecipitation experiments with antibodies targeting endogenous ERLIN2 and FAF2 and could detect EVI/WLS in the pulldown fractions (Supplementary Figure S4C).

© 2021. Published by The Company of Biologists under the terms of the Creative Commons Attribution License (https://creativecommons.org/licenses/by/4.0/).

Figure S4

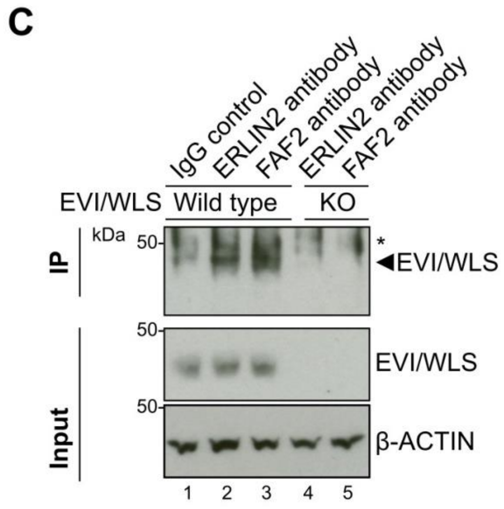

"As WLS expression is known to regulate melanoma cells proliferation (https://doi.org/10.1002/emmm.201201486), please check how knockdown of VCP and UBE2N affect the proliferation (or colony formation) of the melanoma cells."

We tested cell viability and proliferation in A375 cells after candidate gene knock-down and have included the data in Supplementary Figure S5G. In our system and within the time frames relevant for our studies, the knock-down of EVI/WLS did not change melanoma cell proliferation. Despite the increase in EVI/WLS protein levels after siRNA mediated knockdown of UBE2N or VCP, both conditions decrease A375 cell proliferation. We hypothesise that this is due to EVI/WLS-independent effects as both proteins interact with various proteins.

Figure S5

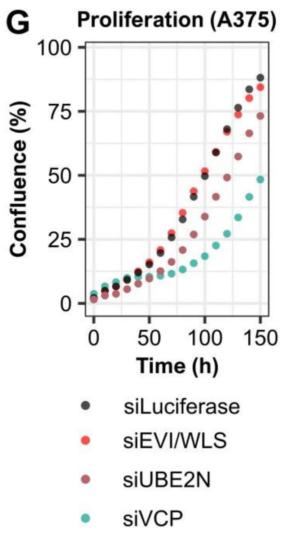

"According to ubibrowser (http://ubibrowser.ncpsb.org/ubibrowser/strict/networkview/networkview/name/Q5T9L3/jobId/ubibrowse-I2020-12-14-98165-1607940581), it is predicted that WLS interacts with different E3 ligases. Please validate whether the knockdown of the E3 ligases expressed in the melanoma cells (such as ITCH) are required for WLS ubiquitination"

We thank the reviewer for the suggestion of this interesting resource. We looked into the predicted E3 ligases and found that all of them have a 'middle' (5) or 'low' (17) confidence

© 2021. Published by The Company of Biologists under the terms of the Creative Commons Attribution License (https://creativecommons.org/licenses/by/4.0/).

interaction score determined by Ubibrowser itself. While they still might be interesting candidates for future studies, most of these proteins seem not to be associated with the ER-membrane or seem not to have been primarily associated with ERAD before. Analysing all of the E3 ligases which are expressed in melanoma cells would be a considerable expansion of our study. Therefore, we only included CHIP/STUB1 into our screening pipeline in HEK293T and A375 melanoma cells, as it has been associated with ERAD before (Lopata et al., 2020, DOI: 10.3390/ijms21155369). Unfortunately, we could not detect an effect on EVI/WLS protein stability in either cell line. The respective data is now included in Figure 1D and Supplementary Figures S1F (HEK293T) and S5E (A375).

Figure 1

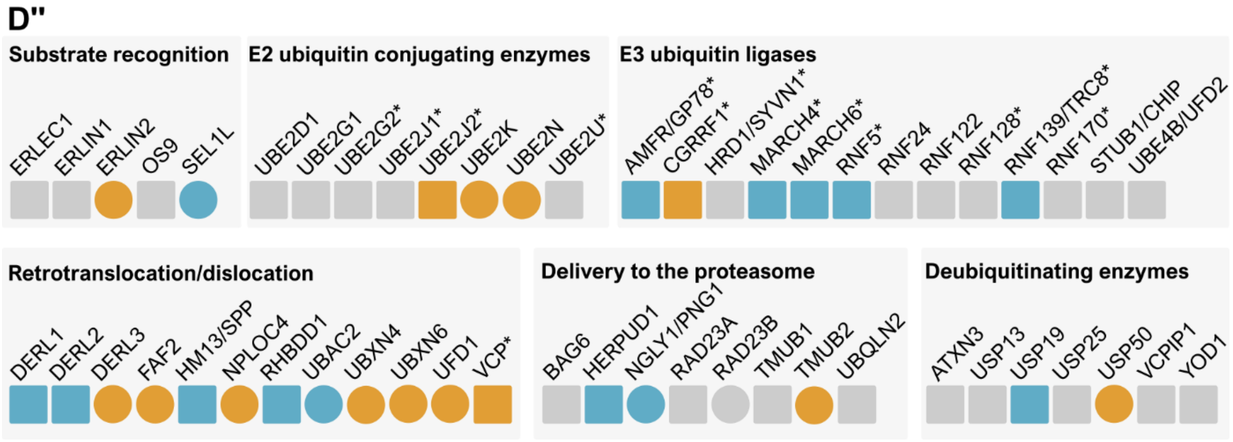

Figure S1

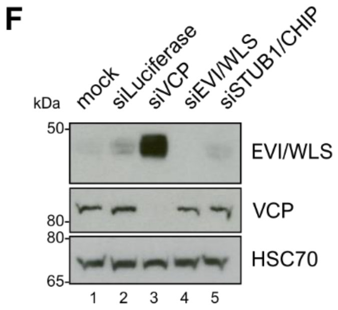

Figure S5

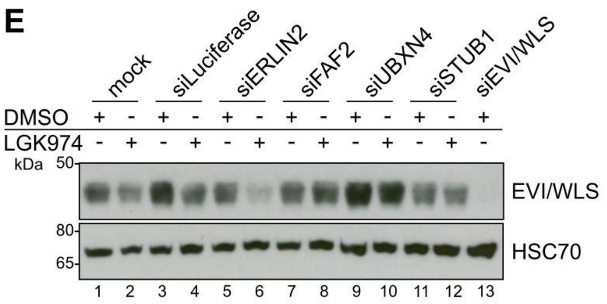

"Minor points:

Please add the statistical analysis to the different qPCR data analyses: Fig 2c, f, i, l etc."

We have added more replicates and a statistical test to the Nano-Luciferase WNT3 secretion data (Figure 4E) and mean and confidence intervals to the different qPCR data analyses.

"I would replace "mini screen" by "low throughput screen" in Supplementary Figure 1."

Thanks for the suggestions, we have changed this accordingly in the revised manuscript.

Reviewer 2

"Advance Summary and Potential Significance to Field:

© 2021. Published by The Company of Biologists under the terms of the Creative Commons Attribution License (https://creativecommons.org/licenses/by/4.0/).

In this study, Wolf et al. identify a number of novel components that are involved in the turnover of the EVI/WLS protein that performs a central role in WNT secretion. Using a well-designed siRNA-based screen, various ERAD-linked proteins are identified that regulate cellular EVI/WLS protein levels. Furthermore, a link is reported between EVI/WLS levels and WNT expression and secretion, indicating that interaction of these proteins mediates their reciprocate stabilisation. Follow-up experiments indicate that the newly identified components regulate EVI/WLS levels via ubiquitination-dependent mechanisms. These results thus shed light on how components of major cellular ubiquitin-mediated degradation pathways jointly operate to control WNT signalling, a key developmental pathway."

Reviewer 2 Comments for the Author:

"In general, data are presented in a clear and comprehensive manner. The screen set-up appears robust and well-controlled, indicating regulation of EVI/WLS at the posttranslational level.

We thank the reviewer for the positive comments.

The follow-up work, aimed at the validation of novel candidates and insight in the underlying mechanisms, however, appears a weaker part of this study. Moreover, data are overinterpreted at a few places. Key points of criticism include a lack of proof for direct ubiquitination of EVI/WLS, and the absence of linking key protein-protein interactions and EVI/WLS degradation steps to subcellular locations. Finally, the consequences of chemical inhibitors with broad cellular impact should be interpreted more carefully."

We thank the reviewer for this critical comment and hope to address the raised concerns accordingly in the revised manuscript.

Specific points:

"Following the first screen in HEK293T cells, candidate genes are evaluated for regulating EVI/WLS levels in A375 cells. However, only previously reported candidates (published) are confirmed here, while newly identified genes are not included. Why not? Demonstration of these observations in 2 cell lines would strengthen the initial findings. (siRNAs are applied in A375 cells in Figure 6, but effects on EVI/WLS levels compared to siLuc are not clear in all cases. Plus, some of these experiments include the use of MG132, which is not an appropriate comparison)"

We have tested the candidates in A375 melanoma cells and included the data in the revised manuscript (Supplementary Figure S5E,F).

Figure S5

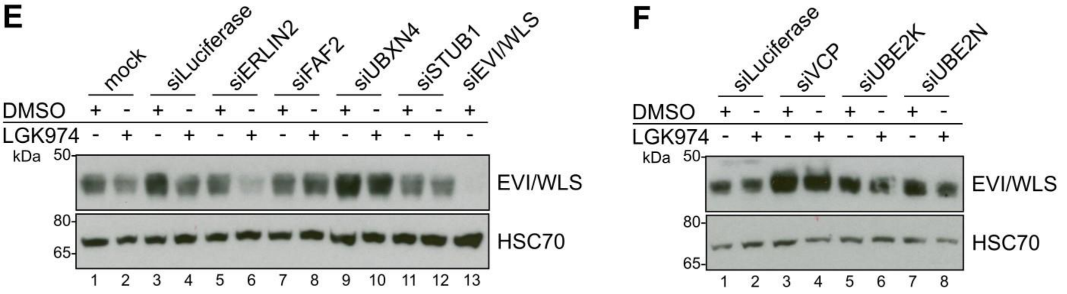

"The manuscript states at multiple places the detection of 'ubiquitinated EVI/WLS', or 'EVI/WLS modified with ubiquitin'. However, evidence for direct modification of the protein is not provided. While the detection of higher MW species for EVI/WLS indeed is a nice lead, it is by no means a direct proof for ubiquitin attachment.

Furthermore, as pulldown experiments (using TUBE or HA-tagged Ub) are seemingly not performed under denaturing conditions, it cannot be excluded that EVI/WLS co-aggregates with other Ub-modified proteins to co-migrate at higher MW regions on the gel.

© 2021. Published by The Company of Biologists under the terms of the Creative Commons Attribution License (https://creativecommons.org/licenses/by/4.0/).

Here, mass spec-based methods would help to demonstrate direct modification of EVI/WLS with ubiquitin. In fact, on page 18 it is mentioned that 'Publicly available MS data report ubiquitination at several lysine residues' (link should be provided), indicating that methods are available"

The reviewer raises an important point and below, we try to explain the lines of evidence we used to conclude that EVI/WLS is indeed directly modified with ubiquitin.

As such, we now emphasised in the main text that SDS-buffer was added to all samples (including pulldowns) before SDS-PAGE and Western blot analyses.

EVI/WLS was detected in a mass-spec-based screen focusing on interactors of ER- associated E3 Ub ligases (Fenech et al., 2020, DOI: 10.7554/eLife.57306) and we could show that it is a substrate of CGRRF1 (Glaeser et al., 2018, DOI: 10.15252/embj.201797311). During the follow-up experiments, DUB treatment was used resulting in reduced high-molecular EVI/WLS bands, again indicating Ub attachment to EVI/WLS (Glaeser et al., 2018, Figure EV3C, DOI: 10.15252/embj.201797311).

Ubiquitin attachment to EVI/WLS was also found in an independent mass-spec analysis recently published on BioRxiv (Supplementary table 4, Steger et al., 2020, https://doi.org/10.1101/2020.07.23.218651).

In addition to this, we have attempted mass-spec experiments to produce additional data to include in the revised manuscript, together with more explanations and better phrasing in the text. However, mass-spec has proven to be difficult for our target protein and given the current situation would require significant more time to adjust the protocols accordingly. Therefore, we believe these experiments would be beyond the scope of this study. It should also be anticipated that not all potential ubiquitination sites are going to be discovered, as ubiquitination on hydroxylated amino acids (potentially mediated by UBE2J2) is frequently lost during standard mass-spec procedures.

"In addition, can the authors express a lysine-less EVI/WLS protein in EVI/WLS-ko cells and demonstrate that this variant is insensitive to the identified siRNAs?"

We thank the reviewer for this suggestion and have generated a lysine-less EVI/WLS construct that also lacks hydroxylated amino acids at positions facing the cytoplasm. It is important to include these, as EVI/WLS can probably be ubiquitinated on serines and threonines by UBE2J2 (Weber et al., 2016, DOI: 10.1016/j.molcel.2016.07.020), which would allow its ubiquitination even in the absence of lysines. Unfortunately, this construct is expressed at much lower levels than the original plasmid that it was derived from. Additionally, this construct still accumulated after knock-down of VCP, indicating that further positions within EVI/WLS (which face the ER lumen) are (also) sufficient for its degradation with the help of VCP. A similar mechanism was described for MHC class I heavy chains by Burr et al., 2013 (DOI: 10.1073/pnas.1303380110). The detailed characterisation of the underlying mechanisms will be subject of future studies in our lab and will also include the data generated on this lysine-less variant. Because this data so far is preliminary, we decided to not include it into this manuscript.

"Page 13: "…the accumulation of K63-ubiquitination of EVI/WLS after MG132 treatment indicates proteasomal degradation." Treatment with proteasomal inhibitors will induce a plethora of changes on overall protein levels and activity, which may cause indirect effects on protein modification and stability, in particular when consequences are evaluated at late timepoints (24h) as indicated in the methods section. Thus, these findings should be interpreted with care. As K63-ubiquitination generally targets for endo-lysosomal turnover of proteins, treatment with bafilomycin, a lysosomal inhibitor, should be included for comparison. In addition, control experiments using a lysine-dead EVI/WLS variant would help to draw more firm conclusions."

We fully agree with this comment and removed this sentence from the revised manuscript. Indeed, treatment with bafilomycin A resulted in increased EVI/WLS protein levels in earlier experiments (Glaeser et al., 2018, Figure EV3A, DOI: 10.15252/embj.201797311).

© 2021. Published by The Company of Biologists under the terms of the Creative Commons Attribution License (https://creativecommons.org/licenses/by/4.0/).

We combined a lysine-less variant of EVI/WLS (EVI/WLS-V5 K410/419R) with a K63-Ub- specific pulldown and detected less ubiquitination on the lysine-less variant when expressed in A375 EVI/WLS knock-out cells. However, the signal was not lost completely, indicating that additional residues might also be important for this type of ubiquitination. We have included the data in Figure 6D of the revised manuscript.

Figure 6

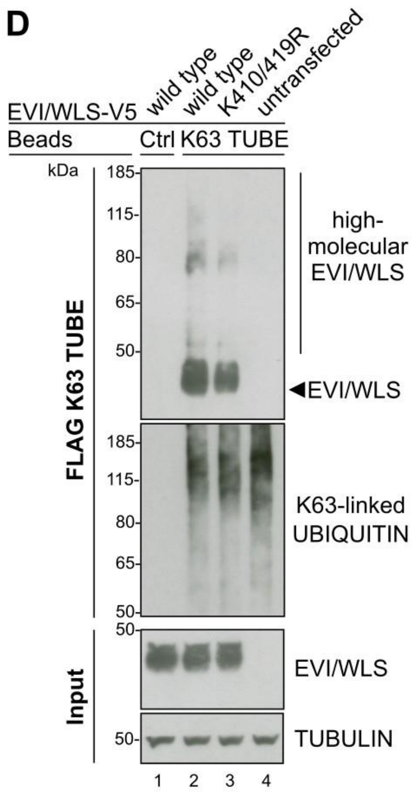

"As the observed effects on EVI/WLS levels likely link to different cellular degradation pathways (qualitative/quantitative ERAD, endo-lysosomal degradation), this point should be better clarified and discussed.

A correlation between key protein-protein interactions and degradation steps with subcellular localization would be required to strengthen this point. Where do the identified components colocalize with EVI/WLS, and where does the EVI/WLS protein accumulate in various knock- down conditions?"

We agree with this comment and expanded the discussion of the revised manuscript accordingly. Furthermore, we performed immunofluorescence stainings to determine where EVI/WLS accumulated after knock-down of UBE2N and to see the colocalization of EVI/WLS, VCP, and FAF2. These experiments were however limited by the availability of antibodies suitable for the endogenous detection of EVI/WLS and its presence and constant shuttling between various cellular compartments. Therefore, we adapted the protocol previously used for EVI/WLS localisation studies by Gasnereau et al. in HELA cells, which relies on the overexpression of a tagged EVI/WLS construct for 24 h (Gasnereau et al., 2011, DOI: 10.1074/jbc.M111.307231). Using this approach in combination with the knock-down of UBE2N (siRNA transfection 72 h before read-out), we could detect a significant increase in corrected total cell fluorescence resulting from the EVI/WLS-V5 plasmid, as expected. To our surprise, this signal remained mostly confined to the ER (Supplementary Figure S6D). We had expected to see accumulation of EVI/WLS in compartments associated with endo-lysosomal degradation after the knock-down of UBE2N. However, co-stainings with markers such as EEA1 (early endosome marker), RAB7 (multivesicular bodies, among others), or lamp1 (lysosome marker, also in combination with Bafilomycin A) did not show colocalization with EVI/WLS-V5.

© 2021. Published by The Company of Biologists under the terms of the Creative Commons Attribution License (https://creativecommons.org/licenses/by/4.0/).

Of course, the interpretation of negative results is always limited and we cannot exclude that EVI/WLS would accumulate in the respective compartments using different study conditions.

Figure S6D

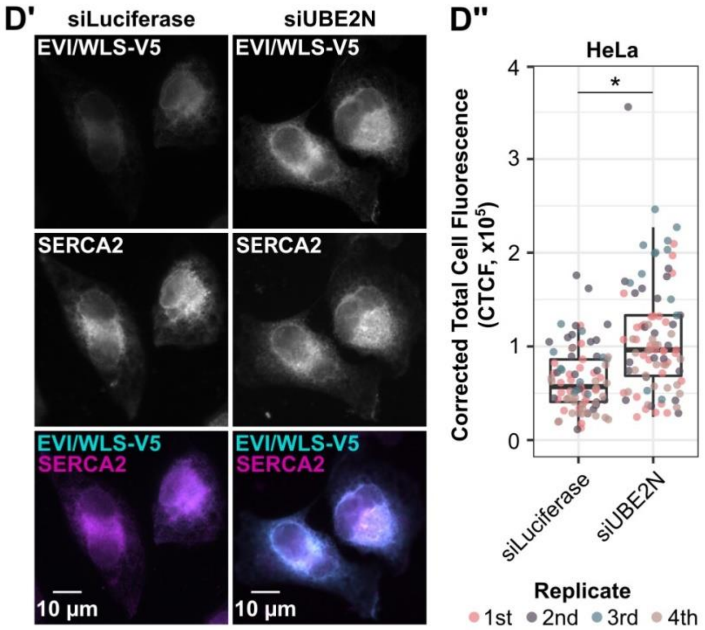

In addition, we also performed colocalization studies using EVI/WLS-V5 and a catalytically dead mutant form of VCP-GFP (VCP-DKO), which traps its substrates and was previously used to confirm EVI/WLS as a VCP substrate (Glaeser et al., 2018, DOI: 10.15252/embj.201797311). These two proteins colocalized with endogenous FAF2 and SERCA2, which was used as an ER marker. We included this data in Supplementary Figure S6B,C).

Figure S6

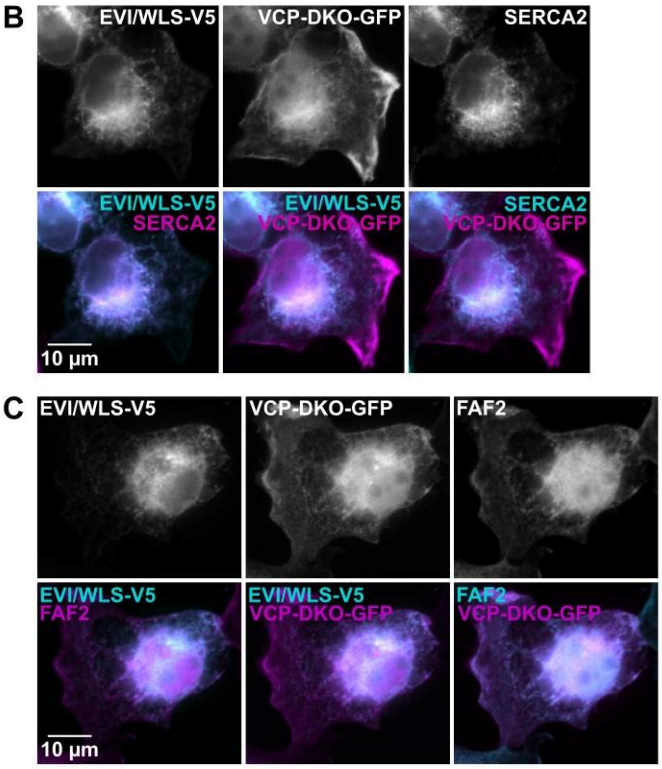

© 2021. Published by The Company of Biologists under the terms of the Creative Commons Attribution License (https://creativecommons.org/licenses/by/4.0/).

Minor points:

"In the results section, a 'focused siRNA and Western blot screen' is mentioned (page 5, top). Here, it would be helpful to provide more information to the reader in the text; which types of genes were screened and what was the rationale?"

Thanks for the suggestions, we added more information in the revised manuscript.

"Figure 1: The DUB enzyme USP50 as well as the proteasome delivery component TMUB2 are also picked up as relevant targets but these are not discussed in the text. What do these findings mean and why were these not discussed or followed up?"

We thank the reviewer for pointing this out. TMUB2 and USP50 as well as other candidates initially chosen were not followed up due to variation in the Western blot experiments between different single siRNAs and biological replicates indicating non- target effects of the siRNAs. The data is presented in the supplementary Figures S2 and S3. We added more details on this in the revised manuscript.

"Reference is made to the phenotype of the UBC13 knockout in c.elegans, where EVI/WLS is diverted to lysosomes (Zhang et al 2018). How does this match with observed increase in EVI/WLS protein levels?"

We thank the reviewer for pointing this out. The exact mechanisms are not clear at this timepoint and from our own localisation experiments (compare Figure S6B,C,D), we would propose that the underlying mechanisms differ from *C. elegans* to humans, as we observe accumulation of EVI/WLS in the ER and not in lysosomes. It is also interesting to note that apparently the lack of K63-linked Ub (and not its presence) lead to lysosomal degradation of MIG-14/EVI/WLS in *C. elegans*. We expanded our discussion on this point.

Typos:
"Abstract: '….regulatory ER-associated degradation (ERAD) has been implicated (in) the production of Wnt Proteins.'
Last sentence of the introduction: '….ERAD, and further emphasises the link between ubiquitination and WNT signalling'"

Thank you. We have revised the manuscript accordingly.

Second decision letter

MS ID#: JOCES/2020/257790

MS TITLE: EVI/WLS function is regulated by ubiquitination and linked to ER-associated degradation by ERLIN2

AUTHORS: Lucie Wolf, Annika Lambert, Julie Haenlin, and Michael Boutros

ARTICLE TYPE: Research Article

I am happy to tell you that your manuscript has been accepted for publication in Journal of Cell Science, pending standard ethics checks.

Reviewer 1

Advance summary and potential significance to field

© 2021. Published by The Company of Biologists under the terms of the Creative Commons Attribution License (https://creativecommons.org/licenses/by/4.0/).

The authors replied to all the comments.

Comments for the author

Please check minor typos:
49 flexible and context-dependent cellular responses to signalling cues besideS various other mechanisms
70 Beside protein quality control, ERAD can also impact (on) cellular signalling by regulating

Reviewer 2

Advance summary and potential significance to field

In this study, Wolf et al. identify a number of novel components that are involved in the turnover of the EVI/WLS protein that performs a central role in WNT secretion. Using a well-designed siRNA-based screen, various ERAD-linked proteins are identified that regulate cellular EVI/WLS protein levels. Follow-up experiments indicate that the newly identified components regulate EVI/WLS levels via ubiquitination-dependent mechanisms. These results thus shed light on how components of major cellular ubiquitin-mediated degradation pathways jointly operate to control WNT signaling, a key developmental pathway.

Comments for the author

The authors have addressed all of my concerns, mostly satisfactorily. I appreciate the transparent response and clear explanations of their findings also of the technical difficulties that were encountered while trying to solve some issues. I think the revised manuscript has much improved and makes a valuable addition to the field.

© 2021. Published by The Company of Biologists under the terms of the Creative Commons Attribution License (https://creativecommons.org/licenses/by/4.0/).
